# Alpha B-Crystallin in Muscle Disease Prevention: The Role of Physical Activity

**DOI:** 10.3390/molecules27031147

**Published:** 2022-02-08

**Authors:** Ivan Dimauro, Daniela Caporossi

**Affiliations:** Unit of Biology and Genetics of Movement, Department of Movement, Human and Health Sciences, University of Rome Foro Italico, Piazza Lauro de Bosis 15, 00135 Rome, Italy; ivan.dimauro@uniroma4.it

**Keywords:** alpha B-crystallin, exercise, muscle diseases

## Abstract

HSPB5 or alpha B-crystallin (CRYAB), originally identified as lens protein, is one of the most widespread and represented of the human small heat shock proteins (sHSPs). It is greatly expressed in tissue with high rates of oxidative metabolism, such as skeletal and cardiac muscles, where HSPB5 dysfunction is associated with a plethora of human diseases. Since HSPB5 has a major role in protecting muscle tissues from the alterations of protein stability (i.e., microfilaments, microtubules, and intermediate filament components), it is not surprising that this sHSP is specifically modulated by exercise. Considering the robust content and the protective function of HSPB5 in striated muscle tissues, as well as its specific response to muscle contraction, it is then realistic to predict a specific role for exercise-induced modulation of HSPB5 in the prevention of muscle diseases caused by protein misfolding. After offering an overview of the current knowledge on HSPB5 structure and function in muscle, this review aims to introduce the reader to the capacity that different exercise modalities have to induce and/or activate HSPB5 to levels sufficient to confer protection, with the potential to prevent or delay skeletal and cardiac muscle disorders.

## 1. Introduction

Organisms, to reduce their susceptibility to various stress conditions such as environmental, metabolic, or pathophysiological stress, have developed adaptive protective mechanisms to maintain or re-establish cellular homeostasis [1]. Strictly dependent on type, intensity and duration, the stressing stimulus can promote either survival with adaptation to adverse conditions or the elimination of excessively damaged cells [2]. The mechanisms of stress response include, among others, a transient modulation of a class of proteins called heat shock proteins (HSPs), highly conserved during evolution [1]. In humans, HSPs display different functions depending on tissue specific localization, intra-or extracellular distribution, developmental expression, and level of induction and interaction with the target protein.

Based on their approximate molecular mass, and as suggested by Kampinga and colleagues (2008) in their new guidelines for the nomenclature of human HSP families, there have been identified five major and broadly conserved families, namely HSPH (Hsp110s), HSPC (Hsp90s), HSPA (Hsp70s), HSPD (Hsp60s), DNAJ (Hsp40s), and small heat shock proteins (sHSPs) [3]. HSPs are known to protect cells at least in part through their chaperone functions. In particular, they facilitate native protein stabilization, translocation, re-folding, and degradation in either an ATP-dependent (large HSPs) or energy independent manner (low weight HSPs) [4,5]. These functions are often performed with the assistance of co-chaperones, which regulate chaperone affinity for each specific substrate. Together, HSPs and their respective co-chaperones ensure protein quality control and protein aggregation, which would be toxic to the cell and lead to programmed cell death or necrosis.

Moreover, HSPs are fundamental for the maintenance of cell structural integrity, interacting with cytoskeletal elements [6]. Therefore, it is not surprising that the expression and activity of HSPs are modulated by physiological and/or pathological stimuli targeting protein stability or homeostasis, including exercise, which recapitulates several stressors such as metabolic disturbances, changes in circulating levels of hormones, increased temperature, as well as induction of mild to severe inflammatory state, and increased production of reactive oxygen and nitrogen species (ROS and RNS) [7,8].

Small heat shock proteins (sHSPs or HSPB) were first discovered as proteins that were upregulated after a heat shock treatment together with several other HSPs, including Hsp70s and Hsp40s [9]. Most eukaryotic genomes contain multiple sHSP genes ranging from 2 in yeast to over 19 in *Arabidopsis thaliana* [10]. They comprise the most widespread but also the most poorly conserved family of molecular chaperones. They show high heterogeneity both in sequence and size [11]. Their common trait is the conserved alpha-crystallin domain, which suggests the presence of only ten chaperones often referred to as HSPB1-HSPB10 (Table 1).

Information on the human HSPB structure and function has mainly been based on cell free in vitro data and on cell biological data with the stress-inducible human HSPB1 and HSPB5 or HSP members from other organisms. These data show that HSPB members can act to block the aggregation of un- or misfolded proteins, and that they can specifically protect cytoskeletal integrity or assist in cytoskeletal recovery upon stress, both functions potentially contributing to the increased survival of cells when exposed to stress conditions that hamper protein homeostasis and/or disrupt the cytoskeleton [12].

The most prominent and well-studied member of sHSPs family is HSPB5 (alpha B-crystallin, CRYAB), a protein playing a crucial role in several cellular processes related to survival and stress recovery [13,14]. HSPB5 is expressed constitutively in a variety of tissues including lens, skeletal muscle, heart, brain, lung, and kidney, as well as in extracellular fluids where it exhibits pleiotropic roles [14,15,16,17,18]. Moreover, scientific evidence highlights the importance of this protein in maintaining cellular functions; indeed, either HSPB5 overexpression or its deleterious mutations are found in a number of known disorders [19,20,21,22,23,24,25,26,27,28,29,30,31,32,33].

Because of the emerging role in physiological and pathological conditions, we aim to summarize in this narrative review what is known about HSPB5 structure and its role in muscle tissue under physiological and pathological conditions. In addition, we report what is known to date about the modulation of HSPB5 following physical activity/exercise and how this response may have the potential to prevent or delay skeletal and cardiac muscle disorders caused by protein misfolding.

## 2. HSPB5 Structure and Regulation

**Figure 1:**
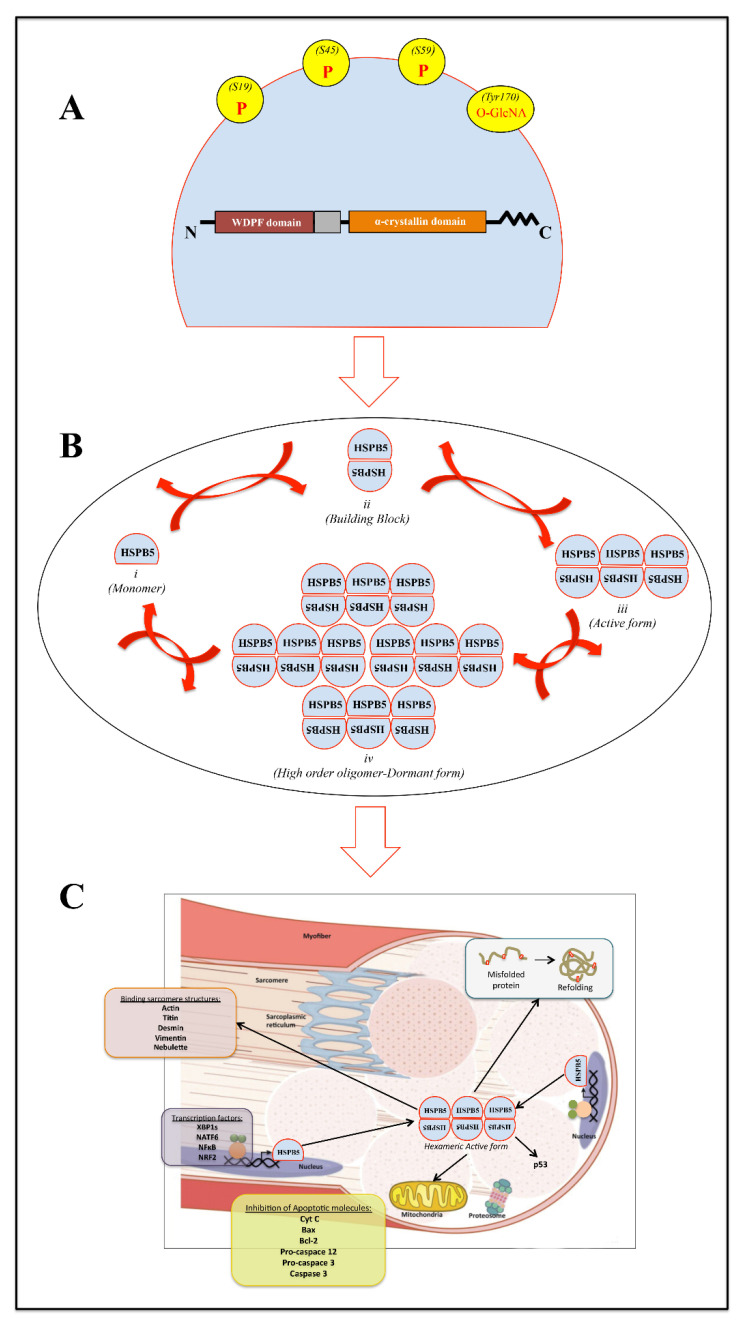
Schematic representation of (**A**) mammalian αB-crystallin protein sequence organization. Gray box: W (tryptophan), D (aspartic acid), P (proline), and F (phenylalanine) (WDPF) amino acid domain; Red bordeaux box: conserved region; orange box: alpha crystallin domain; ΛΛΛΛΛ: flexible domain; P: phosphorylated serine residues; O-GlcNA: O-linked N-acetylglucosamine site at Thr170. (**B**) Exemplified monomer structure (i) of full-length HSPB5. (ii) HSPB5 monomers assemble into dimers (building block) through α-crystallin domain interactions. Higher-order assemblies occur through CTR and the α-crystallin domain to form (iii) hexamers, and poorly defined NTR interactions drive the assembly of the final oligomer (iv). (**C**) Schematic representation of HSPB5 effects/localization within skeletal muscle tissue. HSPB5 is proposed to function at different levels of interrelated cellular pathways, avoiding cytotoxic effects of protein aggregates and apoptosis, as well as preserving sarcomere microstructures such as titin, desmin, actin, vimentin, and nebulette.

The HSPB5 gene is located on chromosome 11 and contains three exons spanning 3.2 kb. The HSPB5 monomeric subunit is a 175 amino acid protein of ~20 kDa, characterized by the presence of a highly conserved central domain, called the “α-crystallin domain (ACD)” (60–150 aa), the flanking N-terminal region (NTR), and the C-terminal region (CTR) [10,11,34] (Figure 1A).

A careful analysis of isolated ACDs highlights a β-sheet sandwich structure composed of eight anti-parallel strands connected by an inter-domain loop. Moreover, it has been demonstrated that these form dimers as basic “building blocks” of higher-order oligomers [35]. In particular, the dimer interface is formed by antiparallel alignment of the β6/β7 strands of the ACD.

Structurally, the CTR domain shows the presence of a motif typically found in many other sHsps and composed of three-residue isoleucine–proline–isoleucine/valine (IXI/V). The secondary structure of an oligomer is called “hexameric block”. It is composed of three HSPB5 dimers, joined through intermolecular interaction between C-terminal IXI/V motif of one dimeric unit and the β4/β8 groove in an ACD of another dimer [36]. Finally, the NTR domain represents the most divergent region among sHsps both in length and sequence [37]. To date, it is clear that the NTR is responsible for the assembly of higher-order HSPB5 oligomers and their dynamic distribution. Indeed, a model of a 24-mer with tetrahedral symmetry can be generated through extensive contacts between NTRs [38,39].

All aforementioned structural models represent a considerable advancement in our understanding of HSPB5 architecture. However, only ~5% of the oligomeric population of HSPB5 exists as a 24-mer [40].

Several studies have also identified short binding sites involved in protein-target interaction in the N-terminal sequence [41], within ACD [42], and a part of the ACD domain of HSPB5 (called “mini-αB-cristallin”) [43], as well as in the C-terminal sequence [44]. This supports the hypothesis that multiple binding sites throughout the molecule act together, presumably in a different manner for different protein targets.

Higher-order HSPB5 oligomers likely represent the dormant storage forms, where the potential substrate-binding sites are engaged in inter-subunit interactions. On the other hand, smaller oligomers exposing hydrophobic patches might contribute, together with dissociated HSPB5 “building blocks”, to the pool of “binding competent” species. Presumably, the transition of HSPB5 from a low- to a high-affinity state occurs through a remodeling of the ensemble composition by adjusting the dissociation/association rates of building blocks, determining the oligomer equilibrium according to the specific needs of the cell (Figure 1B).

An increase in the populations of oligomers with a higher binding capacity, and therefore with an increased chaperone activity, could be induced by conditions that increase the dissociation rate, destabilizing the oligomeric state.

### Post-Translational and Transcriptional HSPB5 Regulation

Serine phosphorylation in HSPB5, as well as in other human sHSPs, appears to promote a shift of its distribution from higher-order oligomers toward smaller species (often tetramers and hexamers) [45]. The three major phosphorylation sites of HSPB5 are serines 19 (S19), 45 (S45), and 59 (S59), all located within the NTR. As already highlighted by Peschek and colleagues [46], NTR contributes decisively to the assembly and dynamics of oligomers and act as a tunable conformation sensor in regulating HSPB5 activity. Therefore, it not surprising that the predominance of small oligomers upon phosphorylation is based on the localization of the phosphorylation sites in NTR and is in accordance with the hierarchical assembly of the oligomers [46].

Depending on the type and/or duration of various stimuli, the fraction of phosphorylated HSPB5 ranges between 10 and 27% [47,48] and can have a dual impact on biological functions: the phosphorylation at initial stage of stress is usually reversible and seems to provide beneficial outcomes, while prolonged stress can induce an irreversible phosphorylation that may lead to deleterious outcomes [49,50].

To date, it is known that MAPKAPK2/3 kinases are responsible for the phosphorylation of S59, while p42/p44 MAPKinase phosphorylates S45 [47]. The specific kinase of S19 is still unknown.

Although it is known that all aforementioned serine residues can be found phosphorylated after various stimuli [51], only a few studies have reported their contemporary involvement in muscle tissues [52,53,54]. Indeed, most of the available data are related to HSPB5 expression and/or activation at Ser59 [55,56,57,58,59,60,61,62,63].

To date, the relationship between the phosphorylation of HSPB5 and its chaperone activity is contradictory. It is hypothesized that the activity upon phosphorylation might depend on the target protein and its interactions [64]. Moreover, Kore and Abraham [65] have highlighted that a majority of the constitutively secreted exosomal HSPB5 is not phosphorylated but present the O-GlcNAc modification on Thr170 in the flexible CTD. The glycosylated and de-phosphorylated HSPB5 then gets selectively packaged into CD63 containing lipid rich multivesicular endosomes, which with the assistance of Rab27 GTPases are then targeted for fusion with the cell membrane and subsequent release of intraluminal vesicles as exosomes.

In mammals, HSPB5 is constitutively expressed in tissues with high rates of oxidative metabolism, including the heart and skeletal muscle [15]. The significance of the constitutive expression of this sHSP is probably linked to protection of the cells against chronic stress or to a specific function in a particular tissue.

**Figure 2:**
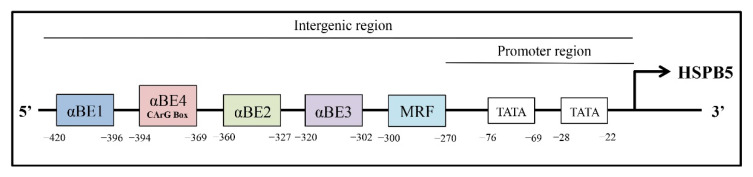
Characterization of HSPB5 upstream enhancers required for the activity of sHSPs. Exon, BE: alpha binding element, MRF: muscle regulatory factor; TATA box. These *cis*-elements localized in the intergenic region of HSPB5 contain the DNA-binding sites or protein binding complexes by which known transcription factors (e.g., AP1, CREB, RORA, AP2F, PAX3) regulate sHSP expression.

The remarkably tissue-specific architectures of the HSPB5 promoters (position and number of the regulatory regions), combined with the tissue-specific expression of various transcriptional regulators, orchestrate gene expression of this sHSP [66]. The developmental expression of HSPB5 in non-lenticular tissues, such as heart, skeletal muscle, kidney, spleen, lung, brain, placenta, and iris, is regulated at the transcriptional level [67,68,69]. Results of DNAase I foot-printing experiments allowed for the characterization of an HSPB5 upstream enhancer required for activity in the non-ocular tissues (position −426/−259), which consists, at least, of 5 *cis*-regulatory elements: 5′-αBE1 (−420/−396), αBE4 (−394/−369), αBE2 (−360/−327), αBE3 (−320/−302), muscle regulatory factor (MRF), (−300/−270)-3′ (Figure 2).

In this regard, the deletion of 87 bp containing the 5′-half of the enhancer (5′-αBE1, αBE4, and half of the αBE2-3′) abrogates the expression of HSPB5 in all tissues [70].

αBE1 is within a region that is essential for glucocorticoid responsiveness [71] and binds the glucocorticoid receptor (GR), thus contributing to the broad expression of HSPB5 [15,72]. The element αBE4 is more specifically required in adult myocardial cells [73] and contains a reverse CArG box (5′-GG(A/T)6CC-3′). This CArG box, which is involved in the regulation of immediate-early genes such as c-fos [74], is bound by a serum response-like factor, whose identity needs to be clarified [73]. However, in 2010, Manukyan et al. showed that in response to various hypertrophic cardiac stimuli, the αBE4 element interacts also with a dynamic complex formed by the transcription factors Nuclear Factor of Activated Cells (NFAT), Nished, and Signal Transducer and Activators of Transcription (STAT-3) [75].

Concerning αBE3 and αBE2, αBE3 is active in all tissues, except in lungs [76], and αBE2 contains an AP2-like binding sequence [77] that requires further investigation.

The MRF element is an E-box (muscle regulatory-binding site), essential in skeletal muscle (C2C12-derived myotubes), adult heart, and, potentially, in lung cells [78]. MyoD or myogenin (or other members from the same family) by binding the E-box in MRF regulates HSPB5 enhancer activity in muscle cells [68], but other elements seem to be required since one E-box is not sufficient for full transcriptional activity [70,79]. Indeed, in primary myocardial cells, MRF is bound by another transcription factor, up-stream-stimulated factor (USF).

Finally, the HSPB5 promoter contains also two TATA sequences: a proximal (−28/−22), allowing the initiation transcription site at the +1 position; and a distal (−76/−69), contributing more to the selective expression in the lens. Mutations of either or both of these sequences in transgenic mice decrease the promoter activity preferentially in the lens compared to the heart or the muscle [80] (Figure 2).

Studies of the inducibility of sHSPs during tissue maintenance/homeostasis and in various stress conditions unraveled different modes of transcriptional regulation, which vary according to the cell systems considered. A number of cis-regulatory elements that regulate the response of HSPB5 to different stresses have been identified, including the heat shock promoter. For instance, following specific stimuli such as heat, oxidative stress, and inflammation, heat shock factor 1 (HSF1), a transcriptional activator, increases the affinity for contiguous repeated pentameric sequences (i.e., nGAAnnTTCnnGAAn) within promoter region called heat shock elements (HSE) through its trimerization [8,58,81,82].

**Figure 3:**
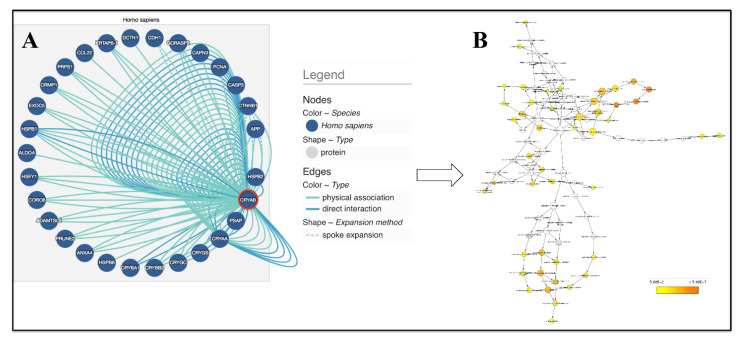
Schematic representation of (**A**) HSPB5 physical association/direct interaction with numerous targets and (**B**) the predominant biological functions resulting from the gene set network. Details are given in Table 2. Biological databases of protein–protein interactions (IntAct), followed by BiNGO, a plug-in of Cytoscape 3.8.2 software are used for the Gene Ontology analysis (*p* < 0.01, only overrepresented categories of biological processes after correction are visualized).

To date, it is interesting to note that through a bioinformatics approach using an open source biological database of protein-protein interactions such as IntAct (last access on 24 January 2022) (http://www.ebi.ac.uk/intact/) and the Biological General Repository for Interaction Datasets (BioGRID) (last access on 21 November 2021) (https://thebiogrid.org/), followed by BiNGO, a Cytoscape plug-in [83,84,85], it is possible to map the physical association/direct interaction of HSPB5 with specific molecular targets (Figure 3A), as well as the predominant biological processes resulting from the gene set network (Figure 3B and Table 2).

In particular, filtering for a MIscore = 1 (range 0–1), where they represent only the interactions with the highest number of experimental evidences supporting that interaction increases, as well as the highest significance level (*p* < 0.01) and only biological processes overrepresented after correction, arise about 28 nodes with a total of 117 edges. Among the numerous biological processes (46 GO ID) modulated by the HSPB5 network, there are the regulation of cell death (e.g., apoptosis), response to stimuli (e.g., heat, abiotic, inorganic substances), protein oligomerization, and multicellular organismal development.

For all readers who are interested in a broader view that encompasses all possible physical interactions of HSPB5 with its numerous targets (161 nodes, 293 edges), as well as their impact on different biological processes, they can consult Supplementary Figure S1 and Table S1.

HSPB5 can also interact with other molecules depending on the type of tissue and stimulus.

## 3. Physiological Roles of HSPB5 in Skeletal and Cardiac Muscle

Many factors, including several members of the sHSP family, such as HSPB5, are known to orchestrate the generation of multinucleated muscle fibers during skeletal muscle differentiation [86]. In particular, the HSPB5 gene contains a skeletal-muscle preferred enhancer (−420 to −270), which includes at least four cis-acting regulatory elements (αBE-1, αBE-2, αBE-3, and MRF) [78,87] (Figure 3), and its protein levels are elevated up to tenfold during skeletal muscle differentiation. Early expression of HSPB5 has also been reported during the developmental phases of the mammalian heart, where it reaches up to 3–5% of the total soluble protein [88]. Unlike from skeletal muscle, the expression of HSPB5 in cardiac tissue requires an additional enhancer such as αBE4 [78] (Figure 2).

In adult skeletal muscle, HSPB5 is associated with the cytoskeletal structures at the level of Z-bands (e.g., actin and titin), and it is highly expressed in slow and fast fibers [23,62,89]. Many different lines of evidence suggest a protective role of this sHSP in mammalian skeletal muscle from heat, oxidative, and mechanical stresses produced during middle age and senescence or by physical activity [58,62,90,91,92,93,94,95,96,97].

In cardiomyocytes, the intracellular localization of HSPB5 has some very unusual characteristics. In particular, data from immunoelectron microscopy has shown that this sHSP localizes in a narrow region of the I-band, rather than in the Z-disc, under both normal and stress conditions.

It has been suggested that the association of HSPB5 with cardiac titin in the N2B region and desmin filaments could either stabilize their conformation or prevent their tendency to form aggregates [63,98,99]. It might be possible that in this tissue, HSPB5 may translocate to the Z-line at an early phase, while a prolonged, irreversible damaging stress leads to extreme stretching of myofibrils and concomitant extension of HSPB5 localization to the I-bands [63,98].

Indeed, the alteration of any of the three major components (i.e., microfilaments, microtubules, and intermediate filaments) induced by different stimuli (e.g., heat shock, oxidative stress, and ischemia) has been demonstrated to induce the phosphorylation of HSPB5 through specific activation of p38MAPK and MAPKAP kinases 2 and 3, as well as its protein synthesis [47,53,58,100,101,102].

The increased level of HSPB5 and its phosphorylation determine, on the one hand, its translocation to the myofilaments where it binds titin, desmin, vimentin, nebulette, and the inactive precursor of caspase 3, leading to the stabilization of the myofilament and to the inhibition of apoptosis [56,103] (Figure 1C); on the other hand, it enhances NFκB activity and its translocation into the nucleus, inducing the expression of genes involved in various biological events such as growth, differentiation, and cell death [56,104,105].

In mammalian cells, during heat shock, oxidative stress, and ischemia, HSPB5 is able to prevent apoptosis by several mechanisms such as the inhibition of RAS-initiated RAF/MEK/ERK signaling pathway [106], or downstream, blocking the BAX, and Bcl-2 translocation from the cytoplasm to the mitochondria [107], as well as interacting with p53 to retain it in the cytoplasm [108], or inhibiting autocatalytic maturation of caspase-3 [109] (Figure 1C).

Finally, a recent finding has established that HSPB5 is necessary for mammalian skeletal muscle homeostasis via modulation of Argonaute 2 (Ago2) activity [57], as well as in cardiomyocytes for the adaptive response to endoplasmic reticulum (ER) stress [110].

## 4. Pathological Roles of HSPB5 in Skeletal and Cardiac Muscle

Given the role of HSPB5 protein in the remodeling of the cytoskeleton during development and cell differentiation, as well as stress conditions, it is not surprising that the myofibrillar myopathy (MFM) of skeletal and cardiac muscle, including desmin-related myopathies (DRM) and dilated (DCM) and restrictive (RCM) cardiomyopathy [21,23,30,111], are caused by mutations of this sHSP gene (Table 3).

The DRM represents a subgroup of myofibrillar myopathy where myopathic manifestations of disease are caused mainly by mutations in desmin or HSPB5 (also known as αB-crystallinopathy) [29,112]. To date, it is known that αB-crystallinopathy is a multisystem disorder characterized by variable combinations of cataracts, cardiomyopathy, myopathy, and progressive muscle weakness affecting the proximal and distal skeletal muscle, respiratory insufficiency, and dysphagia. Cataracts are the most common affection, but myopathies and cardiomyopathies have also been described alone in individuals with mutations in HSPB5, which show either dominant or recessive modes of inheritance and variable penetrance and expressivity.

To date, numerous HSPB5 mutations have been described, but only a few have been shown to have clinical significance in terms of pathogenicity (Table 3).

In 1998, identified for the first time was a family with multisystemic involvement associated with HSPB5 mutation (Arg120Gly) [29]. Vicart and colleagues [29] identified a missense mutation at amino acid position 120 in HSPB5 implicated in the DRM, an autosomal dominant myopathy characterized by weakness of the proximal and distal limb muscles and signs of cardiomyopathy and cataracts.

In the following years, other HSPB5 mutations leading to DRM have been identified, such as Gln151X and Pro155ArgfsTer163 [28], Gly153Ser [26], Ser115ProfsTerf129 [22], Ser21AlafsX24 [20], as well as Asp109 His [27], Asp109Ala [21], and Ala172ProfsTer14 [113], and only recently the mutation c.3G > A, classified as a new type of early-onset MFM [31].

HSPB5 mutations have also been identified in DCM, a disease characterized by cardiac enlargement and systolic dysfunction and often manifested with congestive heart failure [114].

**Table 3:** Mutations described in HSPB5 gene in pathogenic muscle tissues.

Myofibrillar Myopathy	Mutations	Inheritance	Reference
DRM	Ser21AlafsX24	AR	[20]
	Asp109Ala	AD	[21]
	Ser115ProfsTerf129	AR	[22]
	Gly154Ser	AD	[26]
	Asp109 His	AD	[27]
	Gln151X	AD	[28]
	Pro155ArgfsTer163	AD	[28]
	Arg120Gly	AD	[29]
	c.3G > A	AR	[31]
	Ala172ProfsTer14	AD	[113]
DCM	Arg157His	AD	[23]
	Gly154Ser	AD	[25]
	Met1Leu	AR	[30]
	X176Trp	AD	[115]
RCM	Asp109Gly	AD	[111]

DRM: desmin-related myopathies; DCM: dilated cardiomyopathy; RCM: restrictive cardiomyopathy; AD, autosomal dominant; AR: autosomal recessive.

First, Inagaki et al. [23], and then other authors identified several mutations of HSPB5 such as Arg157His [23], Gly154Ser [25], X176Trp [115], and Met1Leu [30]. All these mutations showed different functional alterations from DRM-associated mutation, including the reduced capacity to bind the protein to the cardiac-specific N2B domain, and thereby it may predispose early progression to heart failure under stress conditions.

Finally, RCM-associated mutations have been identified in different genes [116,117,118], including HSPB5 [111]. In particular, Brodehl and colleagues [111] were the first to identify an HSPB5 mutation (Asp109Gly) associated with severe RCM and skeletal myopathy. The presence of cytoplasmic protein aggregates positive for HSPB5 and desmin was indeed associated with Z-band structure partially disappeared in myocardial tissue

## 5. HSPB5 Modulation by Physical Activity/Exercise

In all tissues, physical activity (PA) improves the homeostasis of various macromolecules (DNA, RNA, and proteins) involved in the response to physiological or pathological stress [119,120]. Indeed, the induction of adaptive mechanisms elicited by regular exercise at systemic or at tissue-specific levels has been shown to delay the onset and progression of several diseases and aging-related biomarkers [59,121]. Several studies clearly demonstrated the potential of aerobic and resistance training either to positively improve specific biomarkers related to different pathologies or to reduce the incidence of morbidity and mortality in broad populations of individuals [121]. For example, endurance exercise is well recognized to improve cardiorespiratory fitness, which is an established risk factor for cardiovascular disease and an independent risk factor for type 2 diabetes, while resistance training or aerobic exercises or both improve muscle and cardiorespiratory function and are powerful intervention programs in primary and secondary prevention of muscle diseases [122,123,124,125,126,127].

As already mentioned, results from animal and human studies show that acute or chronic exercise could modulate HSPs in several tissues [127,128]. The changes in HSPs induced by exercise lead to multiple cytoprotective effects on cytoskeleton components, the sarcoplasmic reticulum, and mitochondria [129,130,131], as well as in the maintenance of enzymatic activity, insulin sensitivity, and glucose transport [96,132], and toward apoptosis inhibition [133]. For detailed information on general HSP responses to exercise, readers are directed to other dedicated and very informative reviews [8,128,134].

As highlighted in the previous section, HSPB5 is constitutively highly expressed in both slow and fast fibers of skeletal muscle, as well as in cardiac muscle [63], where it harbors a major role in protecting these tissues from different stressors, possibly leading to alterations of protein stability, particularly the microfilaments, microtubules, and intermediate filament components. Therefore, it is not surprising that several papers have shown its modulation in skeletal muscle from animal and human models by different exercise regimes; however, only a few research results are available with respect to cardiac muscle, all referring to animal models. Table 4 recapitulates the data available so far, with the main information on experimental models, training status/exercise protocols, and HSPB5 modulation.

Since with repetitive exercise, the initial response of some HSPs can be lower as training progresses, the basal levels of HSPB5 content in skeletal muscle seems to be poorly correlated to the training status of human subjects [135,136], with some contradictory results in endurance training, where both increased [137] or decreased [138] HSPB5 content has been reported. However, the acute modulation of HSPB5 after exercise is well documented and seems to be highly dependent on the characteristics of exercise, and specifically on the damaging nature of muscle contraction. Indeed, in non-damaging conditions, such as during concentric contractions determined by endurance exercise, the HSPB5 protein level remain unchanged, independently from the organism or skeletal muscle examined [62,135,138,139,140,141], whereas following an exercise associated with damage to strain-bearing cell structures, HSPB5 expression can be unchanged [95,140,142] or significantly up-regulated [97,140,143,144,145,146,147], possibly depending upon muscle type [148] and training status [147,149].

Regarding the cardiac muscle, it has been shown that the basal level of HSPB5 protein is higher in the heart of rats with low running performance, associated with an increase of other proteins related to stress response [150], while the only paper specifically addressing the exercise-induced modulation of HSPB5 shows that acute endurance exercise does not determine any early upregulation of this protein in mouse heart [63].

Irrespective of the modulation of HSPB5 expression, its phosphorylation in serine 59 and/or translocation from the cytosol to the myofibrillar components or to cellular compartments represent an early response of this small HSP to almost all kinds of acute exercise intervention, both in skeletal muscle and, likely, in cardiac muscle. Usually, the phosphorylated form of HSPB5 is found increased in skeletal muscles with the highest percentage of type I fibers, independently of the type (endurance or resistance) and nature (damaging or not) of exercise [95,143]. Recently, our group demonstrated that the phosphorylation level of HSPB5 induced by an acute bout of non-damaging endurance exercise in mouse is significantly increased only in skeletal muscle tissue with a higher amount of type I and IIA/X myofibers, such as the soleus and red gastrocnemius [62]. Similarly, Jacko and colleagues [141] have shown that, independent of the load volume, a multiple set of resistance exercises in human subjects increases the HSPB5 phosphorylation level in type I myofibers of the vastus lateralis, while it occurs in the type II fibers only after high force demanding loadings. In cardiac muscle, Burniston et al. [151] have demonstrated that the cardiac tissue from rats with low capacity runners exhibits a greater phosphorylation of alpha B-crystallin at serine 59 together with enhanced expression of antioxidant enzymes such as catalase, a common point of convergence in cardiac stress signaling. In line with this evidence, we have recently shown that a single bout of aerobic non-damaging exercise in mice determines an immediate and robust increase of HSPB5 phosphorylation correlated with an increase in lipid peroxidation [63]. These results could reflect the role of HSPB5 in counteracting homeostatic perturbations, including mechanical, thermal, and oxidative stress induced by physical exercise, as well as to reinforce the idea that the phosphorylation of HSPB5 in these tissues possibly reflects the level of stress experienced by the skeletal and cardiac muscle. In this respect, it is worthy of note that, unlike from skeletal muscle, where HSPB5 function and modulation are comparable to those exhibited by HSPB1 [152], in cardiomyocytes, HSPB1 does not show oxidative-induced changes, either at transcriptional, translational, or post-translational modification levels [63,153].

**Table 4:** Exercise-induced modulation of HSPB5 in skeletal and cardiac muscle.

Ref.	Species	Tissues	Tissue Damage	Localization	Type of Exercise *(Sample Collection Timing)*	Level of Analysis	Change
**HUMAN MODELS**							
***RESISTANCE-TYPE OF EXERCISE PROTOCOLS***							
[143]	H	Skeletal muscle (VL)	YES	NS	Acute eccentric exercise *(before and at 1 h and 14 h post-exercise)*	Protein content	↑
[144]	H	Skeletal muscle (VL)	YES	Cytoskeleton vs. cytosol	Acute eccentric exercise *(before and at 30′, 4, 8, 24, 96 h post-exercise)*	mRNA expression Protein content Protein translocation	↑ ↑ ↑
[145]	H	Skeletal muscle (EF)	YES	Cytoskeleton vs. cytosol	Repeated acute eccentric exercise *(before and at 1 h and/or 2, 4, 7 days post-exercise)*	Protein content *(2, 4, 7days)* Protein translocation	↑ ↑
[146]	H	Skeletal muscle (VL)	NS	NS	Acute eccentric exercise *(before and at 5 and 28 h, 5 days post-exercise)*	mRNA expression Protein content	↑ ↑
[148]	H	Skeletal muscle (VL and T)	NS	NS	Strength training *(before and after 2 or 7 weeks of training)*	Protein content *Vastus lateralis* *Trapezius*	↑ =
[149]	H	Skeletal muscle (VL)	YES	NS	Two sessions of acute resistance exercise *(before and at 24 h after the 1st and the 2nd bout)*	mRNA expression Protein content	↑ ↑
[135]	H	Skeletal muscle (VL)	NS	Cytoskeleton vs. cytosol	Acute low-load BFRE or heavy load strength exercise before *(UT)* or after *(TR)* 12-week training *(before and at 1 h post-exercise)*	*UT:* mRNA expression *UT:* Protein translocation *TR:* mRNA expression *TR:* Protein translocation	↑ ↑ = =
[147]	H	Skeletal muscle (VL)	NS	Cytoskeleton vs. cytosol	Acute low-load BFRE or heavy load strength exercise *(before and at 1 h post-exercise)*	mRNA expression Protein content Protein translocation	↑ = ↑
[141]	H	Skeletal muscle (VL)	NS	Type I vs. Type II	Different acute resistance exercise protocols *(before and at 30′ post-exercise)*	Protein phosphorylation Protein translocation	↑ ↑
***ENDURANCE-TYPE OR MIXED-TYPE OF EXERCISE PROTOCOLS***							
[140]	H	Skeletal muscle (VL)	YES	Cytoskeleton vs. cytosol	Acute eccentric exercise *(before and at 3 h post-exercise)*	Protein content Protein translocation Protein phosphorylation	= ↑ ↑
			NO		Acute concentric exercise *(before and at 3 h post-exercise)*	Protein content Protein translocation Protein phosphorylation	= = =
[139]	H	Skeletal muscle (VL)	NO	NS	Acute running exercise *(before and at 24, 48, 72 h post-exercise)*	Protein content	=
[137]	H	Skeletal muscle (VL)	NS	NS	Endurance trained (ET) vs. untrained (U) *(basal values)* Acute endurance exercise in ET *(before and at 48 h and 7 days post-exercise)*	Protein content *ET* vs. *U* *Acute exercise*	↑ =
[138]	H	Skeletal muscle (VL)	NS	Type I vs. Type II	Endurance athletes vs. untrained/resistance athletes	Protein content	↓
[154]	H	Skeletal muscle (VL)	NS	Cytoskeleton vs. cytosol	Endurance training *(before and after training)*	mRNA expression Protein content Protein translocation	= ↓ =
[136]	H	Skeletal muscle (SMT)	NS	NO	Untrained vs. trained (mixed sport disciplines) *(baseline values)*	Protein content	=
[142]	H	Skeletal muscle (VL)	NS	Type I vs. Type II	Endurance (ET) and resistance training (RT, 13 bouts at 8–12 rm in 6 weeks) *(before and 45′ after 1st, 3rd, 7th, 13th bout)*	*ET*: Protein content *ET*: Protein phosphorylation *RT*: Protein content *RT*: Protein phosphorylation	= = = ↑
**ANIMAL MODELS**							
[97]	Rb	Skeletal muscle (TA)	NS	NS	Acute or chronic *(7 or 14 days)* low-frequency motor nerve stimulation *(before and at 0 h post-exercise)*	mRNA expression *Acute protocol* *Chronic protocol*	↑ ↑
[95]	M	Skeletal muscle (EDL)	YES	Cytoskeleton vs. cytosol	Acute lengthening contractions *(before and at 0 h post-exercise)*	Protein content Protein translocation Protein phosphorylation	= ↑ ↑
[62]	M	Skeletal muscle (GR, GW, SOL)	NO	Oxidative vs. glycolytic fibers; Cytoskeleton vs. cytosol	Acute endurance exercise *(before and at 0′, 15′ and 120′ post-exercise)*	mRNA expression Protein content Protein phosphorylation Protein translocation	= = ↑ ↑
[151]	R	Cardiac muscle (heart homogenate)	NS	NS	Low capacity runners vs. high capacity runners *(baseline values)*	Protein phosphorylation	↑
[150]	R	Cardiac muscle(LV)	NS	NO	Low running performance (LRP) vs. high running performance (HRP) *(baseline value)*	Protein content	↑
[63]	M	Cardiac muscle (heart homogenate)	NO	Cytoskeleton vs. cytosol	Acute endurance exercise *(before and at 0′, 15′ and 120′ post-exercise)*	mRNA expression Protein content Protein phosphorylation Protein translocation	= = ↑ ↑

BFRE: blood flow restricted exercise; Rb: rabbit; R: rat; H: human; M: mouse; EDL: extensor digitorum longus; EF: elbow flexor; GR: red gastrocnemius; GW: white gastrocnemius; LV: left ventricle; NS: not specified; SMT: semi tendinous; SOL: soleus; T: trapezius; TA: tibialis anterior; VL: vastus lateralis.

The translocation from the cytosol to specific cellular components is one of the primary steps in the molecular defense system mediated by HSPB5 [49]. In response to acute exercise, the affinity of phosphorylated and non-phosphorylated HSPB5 towards different myofibrillar components increases in both skeletal and cardiac muscle tissue [95,135,140,144,145,147,154], showing specific interaction with β-actin, desmin, and filamin A [62,63].

With respect to the homeostasis perturbation driving the activation of HSPB5 during exercise, it seems clear that, as for all HSPs, also this sHSP must be able to respond quickly to small environmental modifications in order to delay the onset of irreversible protein denaturation [155]. Small variations in temperature, pH, or reactive oxygen species (ROS) concentration can induce HSPB5 activation, all being part of the exercise-induced intracellular changes. Indeed, HSPB5 protein is up-regulated in pathological conditions characterized by elevated oxidative stress, such as desmin-related myopathy, age-induced sarcopenia, and myocardial infarction. In addition, HSPB5 up-regulation plays a pivotal role in skeletal muscle adaptation to the physiological increase of endogenous free radicals during muscle differentiation and contraction [156]. Although strong and compelling evidence in support of a role for HSPB5 in oxidative stress mitigation remains missing, our group demonstrated that “in vitro” exposure of skeletal and cardiac muscle cells to low, non-cytotoxic concentrations of ROS is able to mimic the acute HSPB5 response in skeletal and cardiac muscle tissues to non-damaging exercise “in vivo” [62,63], confirming the contribution of the pro-oxidant environment in HSPB5 phosphorylation and interaction with substrate/client myofibrillar proteins, offering new insights for the study of myofibrillar myopathies and cardiomyopathies.

## 6. Putative Role of Exercise-Induced Modulation of HSPB5 in the Prevention of Muscle Diseases

Given the role of HSPB5 in the remodeling of the cytoskeleton during cell differentiation, development and under stress stimuli, HSPB5 mutations cause several skeletal and cardiac muscle disorders, such as dilated (DCM) and restrictive (RCM) cardiomyopathy, and desmin-related myopathies (DRM) (reviewed in [93]). In these pathologies, the secondary, tertiary, as well as quaternary structures of HSPB5 protein can be compromised, as well as its chaperone activity, with increased or altered substrate affinity leading to the formation of abnormal aggregates, such as the R120G mutation in DRM [157], or the D109G mutation in RCM [111].

Alternatively, HSPB5 mutations can determine the impaired localization of HSPB5 within the cytoskeletal structure, such as the R157H and G154S mutations in DCM, possibly predisposing one to heart failure under stress conditions [23]. Thus, the notion that the structural and functional integrity of HSPB5 plays a pivotal role in preventing proteotoxic-related diseases and in protecting different cells against a wide variety of stresses or pathological insults through its antiapoptotic, anti-inflammatory, and chaperone activity is well-documented [155]. Nevertheless, it is still difficult to clarify if the up-regulation of HSPB5 “per se” has a causative or protective role [158]. Different reports have demonstrated a beneficial effect of HSPB5 up-regulation and/or increased phosphorylation in pathological conditions, including retinal diseases [159], neurodegenerative diseases [160], ischemia/reperfusion [161], and diabetes [162], as well as in aging [163], while a deleterious effect has been described in pulmonary fibrosis [164] and cancer [165]. In skeletal myopathy patients, Unger et al. [166] reported an up-regulation of HSPB5 and HSPB1 (HSP27) in myopathic versus control muscles with the massive binding of HSPB5, HSPB1, and HSPC1 (HSP90) proteins to the sarcomeric I-band region of the altered myofibers. Whereas this interaction could be protective in preventing sarcomeric protein aggregation, the authors also demonstrated that the interaction of the sHSPs with the titin springs could cause the elevated passive tension of human myofibers seen in myopathy.

Noteworthily, both in vitro and in vivo animal studies have demonstrated a clear protective role of HSPB5 in skeletal and cardiac muscle tissues. The pioneering work on this topic was published by Ray et al. in [167], where the authors showed that the hearts of transgenic mice overexpressing HSPB5 displayed a higher functional recovery over a 3 h reperfusion period following a 20 min ischemic period when compared to wild-type mouse hearts. In 2010, we demonstrated that the protection exerted in vitro by vascular endothelial growth factor (VEGF) in murine C2C12 myoblasts toward apoptosis, induced by oxidative or hypoxic-like stress, was clearly linked to the phosphorylation of the KDR/Flk-1 receptor, the activation of NF-κB, and the overexpression of HSPB5 [90]. In the same year, Takagi et al. [168] demonstrated that HSPB5 phosphorylation and translocation from the cytosol to the cytoskeletal fraction was increased in cultured cardiomyocytes exposed to H_2_O_2_ and in murine hearts subjected to ischemia/reperfusion, mediating the cell protective effect exerted in both systems by protein kinase C-related kinase 1 (PRK1 or PKN) activation. Afterwards, various results have indeed verified that depending upon the type of stressors and the degree of induced damage, HSPB5 is modulated at transcriptional and/or post-translational levels in skeletal and cardiac muscle through the different molecular pathways described in the previous section, counteracting myoblasts, myocytes, and cardiomyocytes cell death [56,58,110,161,169,170,171] or stabilizing the cytoskeleton components [62,63,169].

Considering all evidence regarding the elevated concentration and the protective function of HSPB5 in striated muscle tissues, as well as its specific response to muscle loading, there is high expectation for a specific role of exercise-induced HSPB5 modulation in the prevention of diseases caused by protein misfolding [158]. At present, interventional studies in humans are not available. Nevertheless, experimental results from mouse models seem to support this expectation. In the R120G-Tg mouse model of DRM, long-term voluntary (running) exercise reduced the accumulation of pre-amyloid toxic oligomers, paralleled by an increase in lifespan [172].

More recently, the group of Prof. Yamada demonstrated that HSPB5 is specifically involved in the prevention of skeletal muscle weakness induced by non-damaging eccentric training in animal models of rheumatoid arthritis (RA) and idiopathic inflammatory myopathies (IIMs). Specifically, eccentric contractions (ECCs, 4 sets of 5 contractions × 11 sessions) were induced by neuromuscular electrical stimulation (45 V) to the plantar flexor muscles of adjuvant-induced arthritis (AIA) rat, a widely used animal model for RA. The training markedly enhanced the steady-state levels of HSPB5 and its binding to the myofibrils and prevented AIA-induced myofibrillar dysfunction, reduction in contractile proteins, and inflammation–oxidative stress insults [173]. A similar effect has been described in mice with experimental autoimmune myositis, utilized as an IIM animal model. In this protocol, eccentric contractions (20 ECCs every other day for 4 weeks) in electrically-stimulated plantar flexor muscles prevented the decrease in force and the increase in endoplasmic reticulum (ER) stress proteins seen in untrained animals and also enhanced the expression and myofibrillar binding of HSPB1 and HSPPB5 [174].

Although it cannot be excluded that the contraction-induced benefits result from an additive effect of multiple pathways induced by physical exercise, the experimental data collected so far suggest that the modulation of HSPB5 exerted by non-damaging concentric or eccentric muscle contractions may contribute to the protection against skeletal and cardiac muscle weakness by preserving myofibrillar structure and function as well as protecting against oxidative stress insults [175].

## 7. Conclusions and Future Perspective

In addition to the lens, where HSPB5 abundance comes out in favor of a structural function, the high expression of this protein in the heart, where it occupies 3–5% of total soluble protein, and in many other organs, including muscle and brain, points towards a specific role for this heat shock protein, with unique metabolic as well as structural functions. In addition to its chaperon activity in preventing protein aggregation, it is clear that in both skeletal and cardiac tissues, HSPB5 prevents cell death induced by oxidative stress and other cytotoxic stimuli, counteracts the disruption of cytoskeletal assembly, and inhibits inflammation. Moreover, HSPB5 acts in the ubiquitin/proteosome pathway and in cell-cycle progression [176]. Indeed, the data presented in this review demonstrate that a fine-tuning of HSPB5 modulation (i.e., phosphorylation, up- or down-regulation) could represent a new tool to produce beneficial effects under different physio-pathological muscle conditions where this protein is involved.

In several animal models of inflammatory diseases (i.e., multiple sclerosis, stroke, spinal cord injury), the administration of HSPB5 has been found to exert therapeutic effects due to its beneficial chaperone functions [177,178], as well as its ability to trigger protective responses mediated by Toll-like receptor (TLR) 2 in CD14-expressing cells, such as microglia and macrophages [179]. In 2015, a 48-week randomized, placebo-controlled, double-blind Phase IIa trial in relapsing remitting multiple sclerosis (RR-MS) patients showed that repeated intravenous administration of low HSPB5 doses were found to be safe and well tolerated in RR-MS patients and, importantly, led to a progressive decline in MS lesion activity as monitored by magnetic resonance imaging [180].

Skeletal muscle wasting and weakness are consequences of muscular dystrophies and many diseases, such as cancers, disuse, and frailty in ageing. Cardiovascular diseases are still among the most common reasons for mortality and morbidity around the world, and despite major advances in the treatment of cardiac diseases, there is still a great need for treatments capable of counteracting the deterioration of cardiac muscle function in congestive heart failure [181]. The identification of effective interventions that can prevent and/or attenuate the severity of muscle trauma and wasting and restore muscle function are then a priority.

The application of molecular biology techniques to exercise biology has proven a better understanding of the multiplicity and complexity of cellular pathways by which exercise training can prevent or ameliorate the progression of many physio-pathological conditions [182]. In the past, several research groups have shown that regular physical activity can induce a significant enhancement in muscular and functional performance, cardiovascular health, as well as a beneficial anti-aging systemic effect through the modulation of stress-response proteins [122,125,181,183].

In this review, we show how different exercise modalities have the capacity to induce and/or activate HSPB5 to levels sufficient to confer protection to skeletal and cardiac muscle tissues, with the potential to prevent or delay muscle disorders. However, certain questions still remain to be addressed. First, so far, studies on the therapeutic potential of exercise-induced HSPB5 in muscle diseases have been conducted only in rodents. Human studies are now required for effective clinical translation of the research findings from the animal models, at least with respect to skeletal muscle tissues. Different aerobic or resistance exercise protocols have demonstrated their efficacy in patients affected by myositis, such as polymyositis, dermatomyositis, and sporadic inclusion body myositis, in terms of a reduced disease activity and inflammatory markers in muscle biopsies [184,185,186]. Moreover, a randomized control trial to study the effects of high-intensity strength training in patients affected by idiopathic inflammatory myopathies (IIM) has been already approved [187], supporting the possibility of clarifying the role of exercise-induced modulation of HSPB5 in muscle wasting diseases. Second, HSPB5 is generally considered an intracellular protein; however, it has been detected at low levels in extracellular fluids, where it might be released via exosomes under specific stress conditions [16]. Actually, it has been demonstrated that exosomal HSPB5 secreted under stress conditions from astrocytes in a mouse model of multiple sclerosis is able to suppress astrocytes and microglia-mediated inflammatory responses in both autocrine and paracrine manners [188]. Data on the possible modulation of exosomal HSPB5 by contracting muscle tissue are not currently available, and both in vitro and in vivo animal studies are required to verify the role of extracellular HSPB5 in skeletal and cardiac muscle homeostasis.

To conclude, although further studies are recommended to clarify the role of HSPB5 during physiological stimuli, we strongly believe that this knowledge could lead to a better personalization of training protocols with an optimal non-harmful workload in people with different needs and health statuses. In the long term, new knowledge will be expanded so as to accumulate specific insights towards the effectiveness of muscle contraction patterns and the adaptive state of exercise training to be utilized in prevention and rehabilitation.

## Figures and Tables

**Table 1:** Nomenclature of human small heat shock proteins.

Name	Subunit Mol. Mass (kDa)
Hsp27 (HSPB1)	22.8
HSPB2 (MKBP)	20.2
HSPB3	17
αA-crystallin (HSPB4)	19.9
αB-crystallin (HSPB5)	20.2
Hsp20 (HSPB6)	16.8
HSPB7	18.6
Hsp22 (HSPB8, E21G1, α-crystallin C)	21.6
HSPB9 (heat shock protein beta-9, cancer/testis antigen 51 (CT51))	17.5
HSPB10 (outer dense fiber of sperm tails, ODF27, ODFPG, RT7, ODFP, CT133)	28.3

**Table 2:** Enriched biological functions related to HSPB5 network.

GO ID	GO Description	Corrected *p*-Value
43010	camera-type eye development	8.5426 × 10^–9^
2088	lens development in camera-type eye	8.5426 × 10^–9^
1654	eye development	2.8128 × 10^–8^
7423	sensory organ development	1.2632 × 10^–6^
42981	regulation of apoptosis	9.7409 × 10^–6^
43067	regulation of programmed cell death	9.7409 × 10^–6^
10941	regulation of cell death	9.7409 × 10^–6^
48731	system development	2.0836 × 10^–5^
48856	anatomical structure development	6.9007 × 10^–5^
8219	cell death	1.2776 × 10^–4^
16265	death	1.2776 × 10^–4^
7275	multicellular organismal development	2.5032 × 10^–4^
9408	response to heat	2.9070 × 10^–4^
32502	developmental process	6.8889 × 10^–4^
48513	organ development	9.0514 × 10^–4^
60561	apoptosis involved in morphogenesis	9.4592 × 10^–4^
43281	regulation of caspase activity	9.4592 × 10^–4^
9266	response to temperature stimulus	1.0865 × 10^–3^
52548	regulation of endopeptidase activity	1.0865 × 10^–3^
52547	regulation of peptidase activity	1.1177 × 10^–3^
71681	cellular response to indole-3-methanol	1.1177 × 10^–3^
71680	response to indole-3-methanol	1.1177 × 10^–3^
7021	tubulin complex assembly	1.1177 × 10^–3^
43066	negative regulation of apoptosis	1.3476 × 10^–3^
43069	negative regulation of programmed cell death	1.3931 × 10^–3^
60548	negative regulation of cell death	1.5046 × 10^–3^
10035	response to inorganic substance	1.6127 × 10^–3^
9628	response to abiotic stimulus	1.7256 × 10^–3^
10623	developmental programmed cell death	2.4740 × 10^–3^
43065	positive regulation of apoptosis	2.6516 × 10^–3^
43068	positive regulation of programmed cell death	2.6637 × 10^–3^
10942	positive regulation of cell death	2.7111 × 10^–3^
32501	multicellular organismal process	3.3749 × 10^–3^
70306	lens fiber cell differentiation	3.3841 × 10^–3^
50808	synapse organization	3.6119 × 10^–3^
48469	cell maturation	7.2906 × 10^–3^
51259	protein oligomerization	7.2906 × 10^–3^
51402	neuron apoptosis	7.7236 × 10^–3^
46686	response to cadmium ion	7.7236 × 10^–3^
6915	apoptosis	7.7236 × 10^–3^
6916	anti-apoptosis	7.8452 × 10^–3^
12501	programmed cell death	8.0787 × 10^–3^
22402	cell cycle process	8.2637 × 10^–3^
70997	neuron death	8.8151 × 10^–3^
7601	visual perception	9.7040 × 10^–3^
50953	sensory perception of light stimulus	9.7040 × 10^–3^

Biological processes overrepresented after correction. Significance level *p* < 0.01.

## Data Availability

Not applicable.

## Supplementary Materials

The following supporting information can be downloaded, Figure S1: Schematic representation of (A) HSPB5 physical interaction with numerous targets and (B) the predominant biological functions resulting from the gene set network; Table S1: Enriched biological functions related to HSPB5 network.

## References

[1] Kültz D. (2005). Molecular and evolutionary basis of the cellular stress response. Annu. Rev. Physiol..

[2] Fulda S., Gorman A.M., Hori O., Samali A. (2010). Cellular stress responses: Cell survival and cell death. Int. J. Cell Biol..

[3] Kampinga H.H., Hageman J., Vos M.J., Kubota H., Tanguay R.M., Bruford E.A., Cheetham M.E., Chen B., Hightower L.E. (2009). Guidelines for the nomenclature of the human heat shock proteins. Cell Stress Chaperones.

[4] Richter K., Haslbeck M., Buchner J. (2010). The heat shock response: Life on the verge of death. Mol. Cell.

[5] Saibil H. (2013). Chaperone machines for protein folding, unfolding and disaggregation. Nat. Rev. Mol. Cell Biol..

[6] Liang P., MacRae T.H. (1997). Molecular chaperones and the cytoskeleton. J. Cell Sci..

[7] Morton J.P., Kayani A.C., McArdle A., Drust B. (2009). The exercise-induced stress response of skeletal muscle, with specific emphasis on humans. Sports Med..

[8] Dimauro I., Mercatelli N., Caporossi D. (2016). Exercise-induced ROS in heat shock proteins response. Free Radic. Biol. Med..

[9] Karch F., Török I., Tissières A. (1981). Extensive regions of homology in front of the two hsp70 heat shock variant genes in Drosophila melanogaster. J. Mol. Biol..

[10] Kappé G., Franck E., Verschuure P., Boelens W.C., Leunissen J.A.M., de Jong W.W. (2003). The human genome encodes 10 alpha-crystallin-related small heat shock proteins: HspB1-10. Cell Stress Chaperones.

[11] Kriehuber T., Rattei T., Weinmaier T., Bepperling A., Haslbeck M., Buchner J. (2010). Independent evolution of the core domain and its flanking sequences in small heat shock proteins. FASEB J..

[12] Kampinga H.H., Garrido C. (2012). HSPBs: Small proteins with big implications in human disease. Int. J. Biochem. Cell Biol..

[13] Bakthisaran R., Tangirala R., Rao C.M. (2015). Small heat shock proteins: Role in cellular functions and pathology. Biochim. Biophys. Acta.

[14] Thornell E., Aquilina A. (2015). Regulation of αA- and αB-crystallins via phosphorylation in cellular homeostasis. Cell Mol. Life Sci..

[15] Bhat S.P., Nagineni C.N. (1989). alpha B subunit of lens-specific protein alpha-crystallin is present in other ocular and non-ocular tissues. Biochem. Biophys. Res. Commun..

[16] Gangalum R.K., Atanasov I.C., Zhou Z.H., Bhat S.P. (2011). AlphaB-crystallin is found in detergent-resistant membrane microdomains and is secreted via exosomes from human retinal pigment epithelial cells. J. Biol. Chem..

[17] Rothbard J.B., Kurnellas M.P., Brownell S., Adams C.M., Su L., Axtell R.C., Chen R., Fathman C.G., Robinson W.H., Steinman L. (2012). Therapeutic effects of systemic administration of chaperone aB-crystallin associated with binding proinflammatory plasma proteins. J. Biol. Chem..

[18] Li J., Yu J., Xue W., Huang H., Yan L., Sang F., An S., Zhang J., Wang M., Zhang J. (2021). The engineered expression of secreted HSPB5-Fc in CHO cells exhibits cytoprotection in vitro. BMC Biotechnol..

[19] Cornford P.A., Dodson A.R., Parsons K.F., Desmond A.D., Woolfenden A., Fordham M., Neoptolemos J.P., Ke Y., Foster C.S. (2000). Heat shock protein expression independently predicts clinical outcome in prostate cancer. Cancer Res..

[20] Del Bigio M.R., Chudley A.E., Sarnat H.B., Campbell C., Goobie S., Chodirker B.N., Selcen D. (2011). Infantile muscular dystrophy in Canadian aboriginals is an αB-crystallinopathy. Ann. Neurol..

[21] Fichna J.P., Potulska-Chromik A., Miszta P. (2016). A novel dominant D109A CRYAB mutation in a family with myofibrillar myopathy affects αB-crystallin structure. BBA Clin..

[22] Forrest K.M., Al-Sarraj S., Sewry C. (2011). Infantile onset myofibrillar myopathy due to recessive CRYAB mutations. Neuromuscul. Disord..

[23] Inagaki N., Hayashi T., Arimura T., Koga Y., Takahashi M., Shibata H., Teraoka K., Chikamori T., Yamashina A., Kimura A. (2006). Alpha B-crystallin mutation in dilated cardiomyopathy. Biochem. Biophys. Res. Commun..

[24] Liu Y., Zhang X., Luo L., Wu M., Zeng R., Cheng G., Hu B., Liu B., Liang J.J., Shang F. (2006). A novel αB-crystallin mutation associated with autosomal dominant congenital lamellar cataract. Investig. Ophthalmol. Vis. Sci..

[25] Pilotto A., Marziliano N., Pasotti M., Grasso M., Costante A.M., Arbustini E. (2006). alphaB-crystallin mutation in dilated cardiomyopathies: Low prevalence in a consecutive series of 200 unrelated probands. Biochem. Biophys. Res. Commun..

[26] Reilich P., Schoser B., Schramm N., Krause S., Schessl J., Kress W., Müller-Höcker J., Walter M.C., Lochmuller H. (2010). The p. G154S mutation of the Alpha-B crystallin gene (CRYAB) causes late-onset distal myopathy. Neuromuscul. Disord..

[27] Sacconi S., Féasson L., Antoine J.C., Pécheux C., Bernard R., Cobo A.M., Casarin A., Salviati L., Desnuelle C., Urtizberea A. (2012). A novel CRYAB mutation resulting in multisystemic disease. Neuromuscul. Disord..

[28] Selcen D., Engel A.G. (2003). Myofibrillar myopathy caused by novel dominant negative alpha B-crystallin mutations. Ann. Neurol..

[29] Vicart P., Caron A., Guicheney P. (1998). A missense mutation in the alphaB-crystallin chaperone gene causes a desmin-related myopathy. Nat. Genet..

[30] Ma K., Luo D., Tian T., Li N., He X., Rao C., Zhong B., Lu X. (2019). A novel homozygous initiation codon variant associated with infantile alpha-Bcrystallinopathy in a Chinese family. Mol. Genet. Genom. Med..

[31] Lu X.G., Yu U., Han C.X., Mai J.H., Liao J.X., Hou Y.Q. (2021). c.3G>A mutation in the CRYAB gene that causes fatal infantile hypertonic myofibrillar myopathy in the Chinese population. J. Integr. Neurosci..

[32] Oliveira A.O., Osmand A., Outeiro T.F., Muchowski P.J., Finkbeiner S. (2016). αB-Crystallin overexpression in astrocytes modulates the phenotype of the BACHD mouse model of Huntington’s disease. Hum. Mol. Genet..

[33] Chen P., Ji W., Liu F.Y. (2012). Alpha-crystallins and tumorigenesis. Curr. Mol. Med..

[34] Delbecq S.P., Klevit R.E. (2013). One size does not fit all: The oligomeric states of αB crystallin. FEBS Lett..

[35] Bagnéris C., Bateman O.A., Naylor C.E., Cronin N., Boelens W.C., Keep N.H., Slingsby C. (2009). Crystal structures of alpha-crystallin domain dimers of alphaB-crystallin and Hsp20. J. Mol. Biol..

[36] Delbecq S.P., Rosenbaum J.C., Klevit R.E. (2015). A mechanism of subunit recruitment in human small heat shock protein oligomers. Biochemistry.

[37] Kim K.K., Kim R., Kim S.H. (1998). Crystal structure of a small heatshock protein. Nature.

[38] Braun N., Zacharias M., Peschek J., Kastenmüller A., Zou J., Hanzlik M. (2011). Multiple molecular architectures of the eye lens chaperone αB-crystallin elucidated by a triple hybrid approach. Proc. Natl. Acad. Sci. USA.

[39] Jehle S., Vollmar B.S., Bardiaux B., Dove K.K., Rajagopal P., Gonen T., Oschkinat H., Klevita R.E. (2011). N-terminal domain of alphaB-crystallin provides a conformational switch for multimerization and structural heterogeneity. Proc. Natl. Acad. Sci. USA.

[40] Baldwin A.J., Lioe H., Robinson C.V., Kay L.E., Benesch J.L.P. (2011). αBcrystallin polydispersity is a consequence of unbiased quaternary dynamics. J. Mol. Biol..

[41] Sharma K.K., Kumar G.S., Murphy A.S., Kester K. (1998). Identification of 1,1′-bi(4-anilino) naphthalene-5,5′-disulfonic acid binding sequences in alpha-crystallin. J. Biol. Chem..

[42] Ghosh J.G., Estrada M.R., Clark J.I. (2005). Interactive domains for chaperone activity in the small heat shock protein, human alphaB crystallin. Biochemistry.

[43] Banerjee P.R., Pande A., Shekhtman A., Pande J. (2015). Molecular mechanism of the chaperone function of mini-alpha-Crystallin, a 19-residue peptide of human alpha-Crystallin. Biochemistry.

[44] Treweek T.M., Rekas A., Walker M.J., Carver J.A. (2010). A quantitative NMR spectroscopic examination of the flexibility of the C terminal extensions of the molecular chaperones, alphaA- and alphaB-crystallin. Exp. Eye Res..

[45] Rogalla T., Ehrnsperger M., Preville X. (1999). Regulation of Hsp27 oligomerization, chaperone function, and protective activity against oxidative stress/tumor necrosis factor a by phosphorylation. J. Biol. Chem..

[46] Peschek J., Braun N., Rohrberg J., Back K.C., Kriehuber T., Kastenmüller A., Weinkauf S., Buchner J. (2013). Regulated structural transitions unleash the chaperone activity of aB-crystallin. Proc. Natl. Acad. Sci. USA.

[47] Kato K., Ito H., Kamei K., Inaguma Y., Iwamoto I., Saga S. (1998). Phosphorylation of alpha beta-crystallin in mitotic cells and identification enzymatic activities responsible for phosphorylation. J. Biol. Chem..

[48] Eaton P., Fuller W., Bell J.R., Shattock M.J. (2001). AlphaB crystallin translocation and phosphorylation: Signal transduction pathways and preconditioning in the isolated rat heart. J. Mol. Cell Cardiol..

[49] Bakthisaran R., Akula K.K., Tangirala R., Rao C.M. (2016). Phosphorylation of αB-crystallin: Role in stress, aging and patho-physiological conditions. Biochim. Biophys. Acta.

[50] Bartelt-Kirbach B., Wiegreffe C., Birk S., Baur T., Moron M., Britsch S., Golenhofen N. (2021). HspB5/αB-crystallin phosphorylation at S45 and S59 is essential for protection of the dendritic tree of rat hippocampal neurons. J. Neurochem..

[51] Ito H., Okamoto K., Nakayama H., Isobe T., Kato K. (1997). Phosphorylation of alphaB-crystallin in response to various types of stress. J. Biol. Chem..

[52] Reddy V.S., Jakhotia S., Reddy P.Y., Reddy G.B. (2015). Hyperglycemia induced expression, phosphorylation, and translocation of αBcrystallin in rat skeletal muscle. IUBMB Life.

[53] Launay N., Goudeau B., Kato K., Vicart P., Lilienbaum A. (2006). Cell signaling pathways to alphaB-crystallin following stresses of the cytoskeleton. Expt. Cell Res..

[54] Den Engelsman J., Gerrits D., de Jong W.W., Robbins J., Kato K., Boelens W.C. (2005). Nuclear import of {alpha}B-crystallin is phosphorylation-dependent and hampered by hyperphosphorylation of the myopathy-related mutant R120G. J. Biol. Chem..

[55] Ivanov O., Chen F., Wiley E.L. (2008). alphaB-crystallin is a novel predictor of resistance to neoadjuvant chemotherapy in breast cancer. Breast Cancer Res. Treat..

[56] Adhikari A.S., Singh B.N., Rao K.S., Rao C.M. (2011). αB-crystallin, a small heat shock protein, modulates NF-κB activity in a phosphorylationdependent manner and protects muscle myoblasts from TNF-α induced cytotoxicity *Biochim*. Biophys. Acta.

[57] Neppl R.L., Kataoka M., Wang D.Z. (2014). Crystallin-αB regulates skeletal muscle homeostasis via modulation of argonaute2 activity. J. Biol. Chem..

[58] Fittipaldi S., Mercatelli N., Dimauro I., Jackson M.J., Paronetto M.P., Caporossi D. (2015). Alpha B-crystallin induction in skeletal muscle cells under redox imbalance is mediated by a JNK-dependent regulatory mechanism. Free Radic. Biol. Med..

[59] Beltran Valls M.R., Wilkinson D.J., Narici M.V., Smith K., Phillips B.E., Caporossi D., Atherton P.J. (2015). Protein carbonylation and heat shock proteins in human skeletal muscle: Relationships to age and sarcopenia. J. Gerontol. A Biol. Sci. Med. Sci..

[60] Pereira M.B., Santos A.M., Gonçalves D.C. (2015). Corrigendum: αBcrystallin interacts with and prevents stress-activated proteolysis of focal adhesion kinase by calpain in cardiomyocytes. Nat. Commun..

[61] Aggeli I.K., Beis I., Gaitanaki C. (2008). Oxidative stress and calpain inhibition induce alpha B-crystallin phosphorylation via p38-MAPK and calcium signalling pathways in H9c2 cells. Cell Signal..

[62] Dimauro I., Antonioni A., Mercatelli N., Grazioli E., Fantini C., Barone R., Macaluso F., Di Felice V., Caporossi D. (2019). The early response of αB-crystallin to a single bout of aerobic exercise in mouse skeletal muscles depends upon fiber oxidative features. Redox Biol..

[63] Antonioni A., Dimauro I., Fantini C., Barone R., Macaluso F., Di Felice V., Caporossi D. (2020). αB-crystallin response to a pro-oxidant non-cytotoxic environment in murine cardiac cells: An “in vitro” and “in vivo” study. Free Radic. Biol. Med..

[64] Koteiche H.A., McHaourab H.S. (2003). Mechanism of chaperone function in small heat-shock proteins. Phosphorylation-induced activation of two-mode binding in alphaB-crystallin. J. Biol. Chem..

[65] Kore R.A., Abraham E.C. (2016). Phosphorylation negatively regulates exosome mediated secretion of cryAB in glioma cells. Biochim. Biophys. Acta.

[66] De Thonel A., Le Mouël A., Mezger V. (2012). Transcriptional regulation of small HSP-HSF1 and beyond. Int. J. Biochem. Cell Biol..

[67] Dubin R.A., Gopal-Srivastava R., Wawrousek E.F., Piatigorsky J. (1991). Expression of the murine alpha B-crystallin gene in lens and skeletal muscle: Identification of a muscle preferred enhancer. Mol. Cell. Biol..

[68] Gopal-Srivastava R., Piatigorsky J. (1994). Identification of a lens-specific regulatory region (LSR) of the murine alpha B-crystallin gene. Nucleic Acids Res..

[69] Haynes J.I., Duncan M.K., Piatigorsky J. (1996). Spatial and temporal activity of the alpha B-crystallin/small heat shock protein gene promoter in transgenic mice. Dev. Dyn..

[70] Gopal-Srivastava R., Kays W.T., Piatigorsky J. (2000). Enhancer-independent promoter activity of the mouse alphaB-crystallin/small heat shock protein gene in the lens and cornea of transgenic mice. Mech. Dev..

[71] Scheier B., Foletti A., Stark G., Aoyama A., Döbbeling U., Rusconi S., Klemenz R. (1996). Glucocorticoids regulate the expression of the stressprotein alpha B-crystallin. Mol. Cell. Endocrinol..

[72] Dubin R.A., Wawrousek E.F., Piatigorsky J. (1989). Expression of the murine alpha Bcrystallin gene is not restricted to the lens. Mol. Cell. Biol..

[73] Gopal-Srivastava R., Haynes J.I., Piatigorsky J. (1995). Regulation of the murine alpha B-crystallin/small heat shock protein gene in cardiac muscle. Mol. Cell. Biol..

[74] Tuil D., Clergue N., Montarras D., Pinset C., Kahn A., Phan-Dinh-Tuy F. (1990). CC Ar GG boxes, cis-acting elements with a dual specificity. Muscle-specific transcriptional activation and serum responsiveness. J. Mol. Biol..

[75] Manukyan I., Galatioto J., Mascareno E., Bhaduri S., Siddiqui M.A. (2010). Cross-talk between calcineurin/NFAT and Jak/STAT signalling induces cardioprotective alpha B crystallin gene expression in response to hypertrophic stimuli. J. Cell. Mol. Med..

[76] Haynes J.I., Gopal-Srivastava R., Frederikse P.H., Piatigorsky J. (1995). Differential use of the regulatory elements of the alpha B-crystallin enhancer in cultured murine lung (MLg), lens (alpha TN4-1) and muscle (C2C12) cells. Gene.

[77] Imagawa M., Chiu R., Karin M. (1987). Transcription factor AP-2 mediates induction by two different signal-transduction pathways: Protein kinase C and cAMP. Cell.

[78] Gopal-Srivastava R., Piatigorsky J. (1993). The murine alpha B-crystallin/small heat shock protein enhancer: Identification of alpha BE-1, alpha BE-2, alpha BE-3, and MRF control elements. Mol. Cell. Biol..

[79] Weintraub H., Hauschka S., Tapscott S.J. (1991). The MCK enhancer contains a p53 responsive element. Proc. Natl. Acad. Sci. USA.

[80] Haynes J.I., Gopal-Srivastava R., Piatigorsky J. (1997). Alpha B-crystallin TATA sequence mutations: Lens-preference for the proximal TATA box and the distal TATA-like sequence in transgenic mice. Biochem. Biophys. Res. Commun..

[81] Morimoto R.I. (1998). Regulation of the heat shock transcriptional response: Cross talk between a family of heat shock factors, molecular chaperones, and negative regulators. Genes Dev..

[82] Christians E.S., Yan L.J., Benjamin I.J. (2002). Heat shock factor 1 and heat shock proteins: Critical partners in protection against acute cell injury. Crit. Care Med..

[83] Maere S., Heymans K., Kuiper M. (2005). BiNGO: A Cytoscape plugin to assess overrepresentation of gene ontology categories in biological networks. Bioinformatics.

[84] Shannon P., Markiel A., Ozier O., Baliga N.S., Wang J.T., Ramage D., Amin N., Schwikowski B., Ideker T. (2003). Cytoscape: A software environment for integrated models of biomolecular interaction networks. Genome Res..

[85] Hermjakob H., Montecchi-Palazzi L., Lewington C., Mudali S., Kerrien S., Orchard S., Vingron M., Roechert B., Roepstorff P., Valencia A. (2004). IntAct: An open source molecular interaction database. Nucleic Acids Res..

[86] Bucley P.A., Konigsberg I.R. (1974). Myogenic fusion and the duration of the post-mitotic gap (G1). Dev. Biol..

[87] Dubin R.A., Ally A.H., Chung S., Piatigorsky J. (1990). Human alpha Bcrystallin gene and preferential promoter function in lens. Genomics.

[88] Lutsch G., Vetter R., Offhauss U., Wieske M., Gröne H.J., Klemenz R., Schimke I., Stahl J., Benndorf R. (1997). Abundance and location of the small heat shock proteins HSP25 and alphaB-crystallin in rat and human heart. Circulation.

[89] D’Amico D., Fiore R., Caporossi D., Di Felice V.D., Cappello F., Dimauro I., Barone R. (2021). Function and Fiber-Type Specific Distribution of Hsp60 and αB-Crystallin in Skeletal Muscles: Role of Physical Exercise. Biology.

[90] Mercatelli N., Dimauro I., Ciafré S.A., Farace M.G., Caporossi D. (2010). AlphaB-crystallin is involved in oxidative stress protection determined by VEGF in skeletal myoblasts. Free Radic. Biol. Med..

[91] Antinozzi C., Sgrò P., Marampon F., Caporossi D., Del Galdo F., Dimauro I., Di Luigi L. (2021). Sildenafil Counteracts the In Vitro Activation of CXCL-9, CXCL-10 and CXCL-11/CXCR3 Axis Induced by Reactive Oxygen Species in Scleroderma Fibroblasts. Biology.

[92] Brady J.P., Garland D.L., Green D.E., Tamm E.R., Giblin F.J., Wawrousek E.F. (2001). AlphaB-crystallin in lens development and muscle integrity: A gene knockout approach. Investig. Ophthalmol. Vis. Sci..

[93] Dimauro I., Antonioni A., Mercatelli N., Caporossi D. (2018). The role of αB-crystallin in skeletal and cardiac muscle tissues. Cell Stress Chaperones.

[94] Doran P., Gannon J., O’Connell K., Ohlendieck K. (2007). Aging skeletal muscle shows a drastic increase in the small heat shock proteins alphaB-crystallin/HspB5 and cvHsp/HspB7. Eur. J. Cell Biol..

[95] Koh T.J., Escobedo J. (2004). Cytoskeletal disruption and small heat shock protein translocation immediately after lengthening contractions. Am. J. Physiol. Cell Physiol..

[96] Melkani G.C., Cammarato A., Bernstein S.I. (2006). alphaB-crystallin maintains skeletal muscle myosin enzymatic activity and prevents its aggregation under heat-shock stress. J. Mol. Biol..

[97] Neufer P.D., Ordway G.A., Williams R.S. (1998). Transient regulation of c-fos, alpha B-crystallin, and hsp70 in muscle during recovery from contractile activity. Am. J. Physiol..

[98] Golenhofen N., Arbeiter A., Koob R., Drenckhahn D. (2002). Ischemia induced association of the stress protein alpha B-crystallin with I band portion of cardiac titin. J. Mol. Cell Cardiol..

[99] Morrison L.E., Whittaker R.J., Klepper R.E., Wawrousek E.F., Glembotski C.C. (2004). Roles for alphaB-crystallin and HSPB2 in protecting the myocardium from ischemia-reperfusion-induced damage in a KO mouse model. Am. J. Physiol. Heart Circ. Physiol..

[100] Maulik N., Watanabe M., Zu Y.L., Huang C.K., Cordis G.A., Schley J.A., Das D.K. (1996). Ischemic preconditioning triggers the activation of MAP kinases and MAPKAP kinase 2 in rat hearts. FEBS Lett..

[101] Hoover H.E., Thuerauf D.J., Martindale J.J., Glembotski C.C. (2000). Alpha B-crystallin gene induction and phosphorylation by MKK6-activated p38. A potential role for alpha B-crystallin as a target of the p38 branch of the cardiac stress response. J. Biol. Chem..

[102] Morrison L.E., Hoover H.E., Thuerauf D.J., Glembotski C.C. (2003). Mimicking phosphorylation of αB-Crystallin on Serine-59 is necessary and sufficient to provide maximal protection of cardiac myocytes from apoptosis. Circ. Res..

[103] Webster K.A. (2003). Serine phosphorylation and suppression of apoptosis by the small heat shock protein alphaB-crystallin. Circ. Res..

[104] Karin M., Lin A. (2002). NF-kappaB at the crossroads of life and death. Nat. Immunol..

[105] Perkins N.D., Gilmore T.D. (2006). Good cop, bad cop: The different faces of NF-κB. Cell Death Differ..

[106] Li D.W., Liu J.P., Mao Y.W., Xiang H., Wang J., Ma W.Y., Dong Z., Pike H.M., Brown R.E., Reed J.C. (2005). Calcium-activated RAF/MEK/ERK signaling pathway mediates p53-dependent apoptosis and is abrogated by alpha B- crystallin through inhibition of RAS activation. Mol. Biol. Cell.

[107] Mao Y.W., Liu J.P., Xiang H., Li D.W. (2004). Human alphaA- and alphaBcrystallins bind to Bax and Bcl-X(S) to sequester their translocation during staurosporine-induced apoptosis. Cell Death Differ..

[108] Liu S., Li J., Tao Y., Xiao X. (2007). Small heat shock protein alphaBcrystallin binds to p53 to sequester its translocation to mitochondria during hydrogen peroxide induced apoptosis. Biochem. Biophys. Res. Commun..

[109] Kamradt M.C., Chen F., Cryns V.L. (2001). The small heat shock protein alpha B-crystallin negatively regulates cytochrome c- and caspase-8- dependent activation of caspase-3 by inhibiting its autoproteolytic maturation. J. Biol. Chem..

[110] Mitra A., Basak T., Datta K., Naskar S., Sengupta S., Sarkar S. (2013). Role of a-crystallin B as a regulatory switch in modulating cardiomyocyte apoptosis by mitochondria or endoplasmic reticulum during cardiac hypertrophy and myocardial infarction. Cell Death Dis..

[111] Brodehl A., Gaertner-Rommel A., Klauke B., Grewe S.A., Schirmer I., Peterschröder A., Faber L., Vorgerd M., Gummert J., Anselmetti D. (2017). The novel αBcrystallin (CRYAB) mutation p.D109G causes restrictive cardiomyopathy. Hum. Mutat..

[112] Dalakas M.C., Dagvadorj A., Goudeau B., Park K.Y., Takeda K., Simon-Casteras M., Vasconcelos O., Sambuughin N., Shatunov A., Nagle J.W. (2003). Progressive skeletal myopathy, a phenotypic variant of desminmyopathy associated with desmin mutations. Neuromuscul. Disord..

[113] Marcos A.T., Amorós D., Muñoz-Cabello B., Galán F., Rivas Infante E., Alcaraz-Mas L., Navarro-Pando J.M. (2020). A novel dominant mutation in CRYAB gene leading to a severe phenotype with childhood onset. Mol. Genet. Genom. Med..

[114] Richardson P., McKenna W., Bristow M., Maisch B., Mautner B., O’Connell J., Olsen E., Thiene G., Goodwin J., Gyarfas I. (1996). Report of the 1995 World Health Organization/International Society and Federation of Cardiology Task Force on the Definition and Classification of cardiomyopathies. Circulation.

[115] Van der Smagt J.J., Vink A., Kirkels J.H., Nelen M., ter Heide H., Molenschot M.M., Weger R.A., Schellekens P.A., Hoogendijk J., Dooijes D. (2014). Congenital posterior pole cataract and adult onset dilating cardiomyopathy: Expanding the phenotype of αB-crystallinopathies. Clin. Genet..

[116] Arbustini E., Pasotti M., Pilotto A., Pellegrini C., Grasso M., Previtali S., Repetto A., Bellini O., Azan G., Scaffino M. (2006). Desmin accumulation restrictive cardiomyopathy and atrioventricular block associated with desmin gene defects. Eur. J. Heart Fail..

[117] Brodehl A., Ferrier R.A., Hamilton S.J., Greenway S.C., Brundler M.A., Yu W., Gibson W.T., McKinnon M.L., McGillivray B., Alvarez N. (2016). Mutations in FLNC are associated with familial restrictive cardiomyopathy. Hum. Mutat..

[118] Wu W., Lu C.X., Wang Y.N., Liu F., Chen W., Liu Y.T., Han Y.C., Cao J., Zhang S.Y., Zhang X. (2015). Novel Phenotype-Genotype Correlations of Restrictive Cardiomyopathy with Myosin-Binding Protein C (MYBPC3) Gene Mutations Tested by Next-Generation Sequencing. J. Am. Heart Assoc..

[119] Hawley J.A., Hargreaves M., Joyner M.J., Zierath J.R. (2014). Integrative biology of exercise. Cell.

[120] Escobar K.A., Cole N.H., Mermier C.M., VanDusseldorp T.A. (2019). Autophagy and aging: Maintaining the proteome through exercise and caloric restriction. Aging Cell.

[121] Warburton D.E., Nicol C.W., Bredin S.S. (2006). Health benefits of physical activity: The evidence. CMAJ.

[122] Beltran Valls M.R., Dimauro I., Brunelli A., Tranchita E., Ciminelli E., Caserotti P., Duranti G., Sabatini S., Parisi P., Parisi A. (2014). Explosive type of moderate-resistance training induces functional, cardiovascular, and molecular adaptations in the elderly. Age.

[123] Ceci R., Beltran Valls M.R., Duranti G., Dimauro I., Quaranta F., Pittaluga M., Sabatini S., Caserotti P., Parisi P., Parisi A. (2014). Oxidative stress responses to a graded maximal exercise test in older adults following explosive-type resistance training. Redox Biol..

[124] Dimauro I., Scalabrin M., Fantini C., Grazioli E., Beltran Valls M.R., Mercatelli N., Parisi A., Sabatini S., Di Luigi L., Caporossi D. (2016). Resistance training and redox homeostasis: Correlation with age-associated genomic changes. Redox Biol..

[125] Dimauro I., Grazioli E., Lisi V., Guidotti F., Fantini C., Antinozzi C., Sgrò P., Antonioni A., Di Luigi L., Capranica L. (2021). Systemic Response of Antioxidants, Heat Shock Proteins, and Inflammatory Biomarkers to Short-Lasting Exercise Training in Healthy Male Subjects. Oxid. Med. Cell Longev..

[126] Pittaluga M., Sgadari A., Dimauro I., Tavazzi B., Parisi P., Caporossi D. (2015). Physical exercise and redox balance in type 2 diabetics: Effects of moderate training on biomarkers of oxidative stress and DNA damage evaluated through comet assay. Oxid. Med. Cell Longev..

[127] Paronetto M.P., Dimauro I., Grazioli E. (2020). Exercise-mediated downregulation of MALAT1 expression and implications in primary and secondary cancer prevention. Free Radic. Biol. Med..

[128] Henstridge D.C., Febbraio M.A., Hargreaves M. (2016). Heat shock proteins and exercise adaptations. Our knowledge thus far and the road still ahead. J. Appl. Physiol..

[129] Bornman L., Steinmann C.M.L., Gericke G.S., Polla B.S. (1998). In vivo heat shock protects rat myocardial mitochondria. Biochem. Biophys. Res. Commun..

[130] Sammut I.A., Harrison J.C. (2003). Cardiac mitochondrial complex activity is enhanced by heats hock proteins. Clin. Exp. Pharm. Phys..

[131] Tupling A.R., Gramolini A.O., Duhamel T.A., Kondo H., Asahi M., Tsuchiya S.C., Borrelli M.J., Lepock J.R., Otsu K., Hori M. (2004). HSP70 binds to the fast-twitch skeletal muscle sarco(endo)plasmic reticulum Ca2þ-ATPase(SERCA1a) and prevents thermal inactivation. J. Biol. Chem..

[132] Chung J., Nguyen A.K., Henstridge D.C., Holmes A.G., Chan M.H.S., Mesa J.L., Lancaster G.I., Southgate R.J., Bruce C.R., Duffy S.J. (2008). HSP72 protects against obesity-induced insulin resistance. Proc. Natl. Acad. Sci. USA.

[133] Gabai V.L., Sherman M.Y. (2002). Interplay between molecular chaperones and signaling pathways in survival of heat shock. J. Appl. Physiol..

[134] Noble E.G., Milne K.J., Melling C.W. (2008). Heat shock proteins and exercise: A primer. Appl. Physiol. Nutr. Metab..

[135] Cumming K.T., Paulsen G., Wernbom M., Ugelstad I., Raastad T. (2014). Acute response and subcellular movement of HSP27, αB-crystallin and HSP70 in human skeletal muscle after blood-flow-restricted low-load resistance exercise. Acta Physiol..

[136] Magi F., Dimauro I., Margheritini F., Duranti G., Mercatelli N., Fantini C., Ripani F.R., Sabatini S., Caporossi D. (2018). Telomere length is independently associated with age, oxidative biomarkers, and sport training in skeletal muscle of healthy adult males. Free Radic. Res..

[137] Morton J.P., Maclaren D.P., Cable N.T., Campbell I.T., Evans L., Kayani A.C., McArdle A., Drust B. (2008). Trained men display increased basal heat shock protein content of skeletal muscle. Med. Sci. Sports Exerc..

[138] Folkesson M., Mackey A.L., Langberg H., Oskarsson E., Piehl-Aulin K., Henriksson J., Kadi F. (2013). The expression of heat shock protein in human skeletal muscle: Effects of muscle fibre phenotype and training background. Acta Physiol..

[139] Morton J.P., MacLaren D.P., Cable N.T., Bongers T., Griffiths R.D., Campbell I.T., Evans L., Kayani A., McArdle A., Drust B. (2006). Time course and differential responses of the major heat shock protein families in human skeletal muscle following acute nondamaging treadmill exercise. J. Appl. Physiol..

[140] Frankenberg N.T., Lamb G.D., Overgaard K., Murphy R.M., Vissing K. (2014). Small heat shock proteins translocate to the cytoskeleton in human skeletal muscle following eccentric exercise independently of phosphorylation. J. Appl. Physiol..

[141] Jacko D., Bersiner K., Hebchen J., de Marées M., Bloch W., Gehlert S. (2019). Phosphorylation of αB-crystallin and its cytoskeleton association differs in skeletal myofiber types depending on resistance exercise intensity and volume. J. Appl. Physiol..

[142] Jacko D., Bersiner K., Schulz O., Przyklenk A., Spahiu F., Höhfeld J., Bloch W., Gehlert S. (2020). Coordinated alpha-crystallin B phosphorylation and desmin expression indicate adaptation and deadaptation to resistance exercise-induced loading in human skeletal muscle. Am. J. Physiol. Cell Physiol..

[143] Féasson L., Stockholm D., Freyssenet D., Richard I., Duguez S., Beckmann J.S., Denis C. (2002). Molecular adaptations of neuromuscular disease-associated proteins in response to eccentric exercise in human skeletal muscle. J. Physiol..

[144] Paulsen G., Vissing K., Kalhovde J.M., Ugelstad I., Bayer M.L., Kadi F., Schjerling P., Hallén J., Raastad T. (2007). Maximal eccentric exercise induces a rapid accumulation of small heat shock proteins on myofibrils and a delayed HSP70 response in humans. Am. J. Physiol. Regul. Integr. Comp. Physiol..

[145] Paulsen G., Lauritzen F., Bayer M.L., Kalhovde J.M., Ugelstad I., Owe S.G., Hallén J., Bergersen L.H., Raastad T. (2009). Subcellular movement and expression of HSP27, alphaB-crystallin, and HSP70 after two bouts of eccentric exercise in humans. J. Appl. Physiol..

[146] Mikkelsen U.R., Paulsen G., Schjerling P., Helmark I.C., Langberg H., Kjær M., Heinemeier K.M. (2013). The heat shock protein response following eccentric exercise in human skeletal muscle is unaffected by local NSAID infusion. Eur. J. Appl. Physiol..

[147] Cumming K.T., Ellefsen S., Rønnestad B.R., Ugelstad I., Raastad T. (2017). Acute and long-term effects of blood flow restricted training on heat shock proteins and endogenous antioxidant systems. Scand. J. Med. Sci. Sports.

[148] Paulsen G., Hanssen K.E., Rønnestad B.R., Kvamme N.H., Ugelstad I., Kadi F., Raastad T. (2012). Strength training elevates HSP27, HSP70 and αB-crystallin levels in musculi vastus lateralis and trapezius. Eur. J. Appl. Physiol..

[149] Murton A.J., Billeter R., Stephens F.B., Des Etages S.G., Graber F., Hill R.J., Marimuthu K., Greenhaff P.L. (2014). Transient transcriptional events in human skeletal muscle at the outset of concentric resistance exercise training. J. Appl. Physiol..

[150] Ribeiro L.P., Freitas-Lima L.C., Naumann G.B., Meyrelles S.S., Lunz W., Pires S.F., Andrade H.M., Carnielli J.B.T., Figueiredo S.G. (2018). Cardiac protein expression patterns are associated with distinct inborn exercise capacity in non-selectively bred rats. Braz. J. Med. Biol. Res..

[151] Burniston J.G., Kenyani J., Wastling J.M., Burant C.F., Qi N.R., Koch L.G., Britton S.L. (2011). Proteomic analysis reveals perturbed energy metabolism and elevated oxidative stress in hearts of rats with inborn low aerobic capacity. Proteomics.

[152] Mehlen P., Kretz-Remy C., Préville X., Arrigo A.P. (1996). Human hsp27, Drosophila hsp27 and human alphaB-crystallin expression-mediated increase in glutathione is essential for the protective activity of these proteins against TNFalpha-induced cell death. EMBO J..

[153] White M.Y., Hambly B.D., Jeremy R.W., Cordwell S.J. (2006). Ischemia-specific phosphorylation and myofilament translocation of heat shock protein 27 precedes alpha B-crystallin and occurs independently of reactive oxygen species in rabbit myocardium. J. Mol. Cell Cardiol..

[154] Cumming K.T., Raastad T., Holden G., Bastani N.E., Schneeberger D., Paronetto M.P., Mercatelli N., Ostgaard H.N., Ugelstad I., Caporossi D. (2014). Effects of vitamin C and E supplementation on endogenous antioxidant systems and heat shock proteins in response to endurance training. Physiol. Rep..

[155] Hayashi J., Carver J.A. (2020). The multifaceted nature of αB-crystallin. Cell Stress Chaperones.

[156] Fittipaldi S., Dimauro I., Mercatelli N., Caporossi D. (2014). Role of exercise-induced reactive oxygen species in the modulation of heat shock protein response. Free Radic. Res..

[157] Michiel M., Skouri-Panet F., Duprat E., Simon S., Férard C., Tardieu A., Finet S. (2009). Abnormal assemblies and subunit exchange of alphaB-crystallin R120 mutants could be associated with destabilization of the dimeric substructure. Biochemistry.

[158] Reddy V.S., Reddy G.B. (2015). Emerging role for αB-crystallin as a therapeutic agent: Pros and cons. Curr. Mol. Med..

[159] Thanos S., Bohm M.R., Meyer zu Horste M., Prokosch-Willing V., Hennig M., Bauer D., Heiligenhaus A. (2014). Role of crystallins in ocular neuroprotection and axonal regeneration. Prog. Retin. Eye Res..

[160] Zhu Z., Reiser G. (2018). The small heat shock proteins, especially HspB4 and HspB5 are promising protectants in neurodegenerative diseases. Neurochem. Int..

[161] Cubedo J., Vilahur G., Casaní L., Mendieta G., Gómez-Jabalera E., Juan-Babot O., Padró T., Badimon L. (2016). Targeting the molecular mechanisms of ischemic damage: Protective effects of alpha-crystallin-B. Int. J. Cardiol..

[162] Reddy V.S., Kumar C.U., Reddy G.B. (2014). Effect of chronic hyperglycemia on crystallin levels in rat lens. Biochem. Biophys. Res. Commun..

[163] Lim E.F., Musa A., Frederick A., Ousman S.S. (2017). AlphaB-crystallin expression correlates with aging deficits in the peripheral nervous system. Neurobiol. Aging.

[164] Bellaye P.S., Wettstein G., Burgy O., Besnard V., Joannes A., Colas J., Causse S., Marchal-Somme J., Fabre A., Crestani B. (2014). The small heat-shock protein αB-crystallin is essential for the nuclear localization of Smad4: Impact on pulmonary fibrosis. J. Pathol..

[165] Yang M., Li Y., Tian F. (2021). Association between Alpha B-crystallin expression and prognosis in patients with solid tumors: A protocol for systematic review and meta-analysis. Medicine.

[166] Unger A., Beckendorf L., Böhme P., Kley R., von Frieling-Salewsky M., Lochmüller H., Schröder R., Fürst D.O., Vorgerd M., Linke W.A. (2017). Translocation of molecular chaperones to the titin springs is common in skeletal myopathy patients and affects sarcomere function. Acta Neuropathol. Commun..

[167] Ray P.S., Martin J.L., Swanson E.A., Otani H., Dillmann W.H., Das D.K. (2001). Transgene overexpression of alphaB crystallin confers simultaneous protection against cardiomyocyte apoptosis and necrosis during myocardial ischemia and reperfusion. FASEB J..

[168] Takagi H., Hsu C.P., Kajimoto K., Shao D., Yang Y., Maejima Y., Zhai P., Yehia G., Yamada C., Zablocki D. (2010). Activation of PKN mediates survival of cardiac myocytes in the heart during ischemia/reperfusion. Circ. Res..

[169] Chis R., Sharma P., Bousette N., Miyake T., Wilson A., Backx P.H., Gramolini A.O. (2012). Crystallin B prevents apoptosis after H2O2 exposure in mouse neonatal cardiomyocytes. Am. J. Physiol. Heart Circ. Physiol..

[170] Kötter S., Unger A., Hamdani N., Lang P., Vorgerd M., Nagel-Steger L., Linke W.A. (2014). Human myocytes are protected from titin aggregation-induced stiffening by small heat shock proteins. J. Cell. Biol..

[171] Maloyan A., Gulick J., Glabe C.G., Kayed R., Robbins J. (2007). Exercise reverses preamyloid oligomer and prolongs survival in alphaB-crystallin-based desmin-related cardiomyopathy. Proc. Natl. Acad. Sci. USA.

[172] Bhuiyan M.S., Pattison J.S., Osinska H., James J., Gulick J., McLendon P.M., Hill J.A., Sadoshima J., Robbins J. (2013). Enhanced autophagy ameliorates cardiac proteinopathy. J. Clin. Investig..

[173] Himori K., Tatebayashi D., Ashida Y., Yamada T. (2019). Eccentric training enhances the αB-crystallin binding to the myofibrils and prevents skeletal muscle weakness in adjuvant-induced arthritis rat. J. Appl. Physiol..

[174] Himori K., Ashida Y., Tatebayashi D., Abe M., Saito Y., Chikenji T., Westerblad H., Andersson D.C., Yamada T. (2021). Eccentric Resistance Training Ameliorates Muscle Weakness in a Mouse Model of Idiopathic Inflammatory Myopathies. Arthritis Rheumatol..

[175] Christians E.S., Ishiwata T., Benjamin I.J. (2012). Small Heat Shock Proteins in Redox Metabolism: Implications for Cardiovascular Diseases. Int. J. Biochem. Cell Biol..

[176] Garrido C., Paul C., Seigneuric R., Kampinga H.H. (2012). The small heat shock proteins family: The long forgotten chaperones. Int. J. Biochem. Cell Biol..

[177] Ousman S.S., Tomooka B.H., van Noort J.M., Wawrousek E.F., O’Connor K.C., Hafler D.A., Sobel R.A., Robinson W.H., Steinman L. (2007). Protective and therapeutic role for alpha B-crystallin in autoimmune demyelination. Nature.

[178] Arac S.E., Brownell J.B., Rothbard C., Chen R.M., Ko M.P., Pereira G.W., Albers G.W., Steinman L., Steinberg G.K. (2011). Systemic augmentation of alpha B-crystallin provides therapeutic benefit twelve hours post-stroke onset via immune modulation. Proc. Natl. Acad. Sci. USA.

[179] Van Noort J.M., Bsibsi M., Nacken P.J., Gerritsen W.H., Amor S., Holtman I.R., Boddeke E., van Ark I., Leusink-Muis T., Folkerts G. (2013). Activation of an immune-regulatory macrophage response and inhibition of lung inflammation in a mouse model of COPD using heat-shock protein alpha B-crystallin-loaded PLGA microparticles. Biomaterials.

[180] Van Noort J.M., Bsibsi M., Nacken P.J., Verbeek R., Venneker E.H. (2015). Therapeutic Intervention in Multiple Sclerosis with Alpha B-Crystallin: A Randomized Controlled Phase IIa Trial. PLoS ONE.

[181] Tarone G., Brancaccio M. (2014). Keep your heart in shape: Molecular chaperone networks for treating heart disease. Cardiovasc. Res..

[182] Neufer P.D., Bamman M.M., Muoio D.M., Bouchard C., Cooper D.M., Goodpaster B.H., Booth F.W., Kohrt W.M., Gerszten R.E., Mattson M.P. (2015). Understanding the Cellular and Molecular Mechanisms of Physical Activity-Induced Health Benefits. Cell Metab..

[183] Thakur S.S., Swiderski K., Ryall J.G., Lynch G.S. (2018). Therapeutic potential of heat shock protein induction for muscular dystrophy and other muscle wasting conditions. Philos. Trans. R. Soc. Lond. B Biol. Sci..

[184] Varjú C., Pethö E., Kutas R., Czirják L. (2003). The effect of physical exercise following acute disease exacerbation in patients with dermato/polymyositis. Clin. Rehabil..

[185] Munters L.A., Loell I., Ossipova E., Raouf J., Dastmalchi M., Lindroos E., Chen Y.W., Esbjörnsson M., Korotkova M., Alexanderson H. (2016). Endurance Exercise Improves Molecular Pathways of Aerobic Metabolism in Patients with Myositis. Arthritis Rheumatol..

[186] Jørgensen A.N., Aagaard P., Frandsen U., Boyle E., Diederichsen L.P. (2018). Blood-flow restricted resistance training in patients with sporadic inclusion body myositis: A randomized controlled trial. Scand. J. Rheumatol..

[187] Jensen K.Y., Aagaard P., Schrøder H.D., Suetta C., Nielsen J.L., Boyle E., Diederichsen L.P. (2021). High-intensity strength training in patients with idiopathic inflammatory myopathies: A randomised controlled trial protocol. BMJ Open.

[188] Guo Y.S., Liang P.Z., Lu S.Z., Chen R., Yin Y.Q., Zhou J.W. (2019). Extracellular αB-crystallin modulates the inflammatory responses. Biochem. Biophys. Res. Commun..

