# Supplementary material for: Alpha B-Crystallin in Muscle Disease Prevention: The Role of Physical Activity
Source: Molecules. 2022 Feb 8;27(3):1147. doi: 10.3390/molecules27031147 (PMC8840510; doi:10.3390/molecules27031147)
Supplement: Supplementary file 1 [file molecules-27-01147-s001.zip › molecules-1544390-supplementary.pdf]

## Supplementary Materials

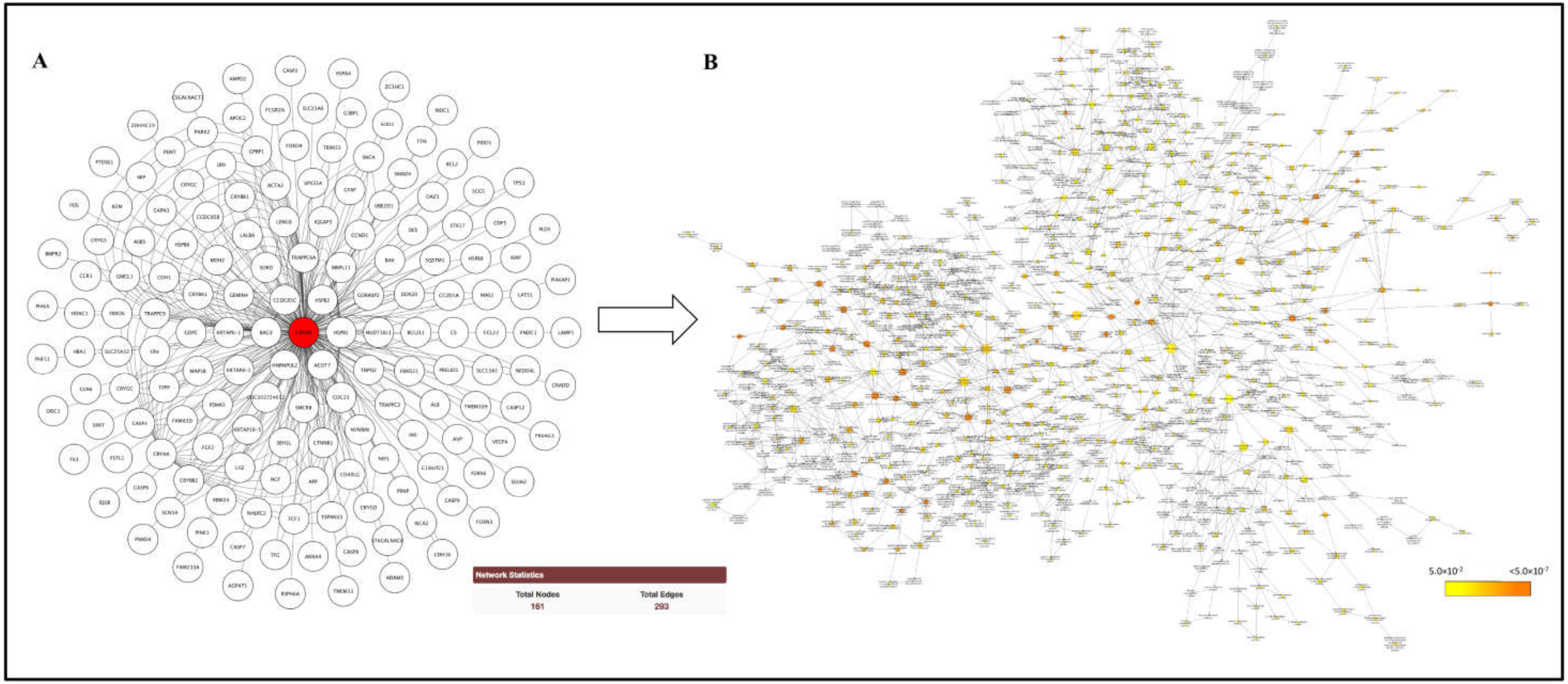


**Figure S1.** Schematic representation of (**A**) HSPB5 physical interaction with numerous targets and (**B**) the predominant biological functions resulting from the gene set network. All details are given in Supplementary Table S1. General Repository for Interaction Datasets (BioGRID), followed by BiNGO, a plug-in of Cytoscape 3.8.2 software were used for the Gene Ontology analysis ($p < 0.05$, all biological processes are visualized).

**Table S1.** Enriched biological functions related to HSPB5 network.

| GO ID | GO Description | Corrected *p*-Value |
|---|---|---|
| **10941** | regulation of cell death | 1.3106E–13 |
| **43067** | regulation of programmed cell death | 3.3024E–13 |
| **42981** | regulation of apoptosis | 1.0456E–12 |
| **60548** | negative regulation of cell death | 1.3226E–12 |
| **43066** | negative regulation of apoptosis | 3.1712E–10 |
| **10035** | response to inorganic substance | 3.1712E–10 |
| **43069** | negative regulation of programmed cell death | 3.1712E–10 |
| **9628** | response to abiotic stimulus | 9.3241E–10 |
| **6916** | anti-apoptosis | 1.2442E–9 |
| **9266** | response to temperature stimulus | 1.5475E–9 |
| **48522** | positive regulation of cellular process | 1.0653E–8 |
| **9408** | response to heat | 7.2713E–8 |
| **48518** | positive regulation of biological process | 8.0915E–8 |
| **2088** | lens development in camera-type eye | 1.2106E–7 |
| **31399** | regulation of protein modification process | 2.1695E–7 |
| **48523** | negative regulation of cellular process | 2.4370E–7 |
| **32270** | positive regulation of cellular protein metabolic process | 2.4370E–7 |
| **10038** | response to metal ion | 2.4774E–7 |
| **32268** | regulation of cellular protein metabolic process | 2.5105E–7 |
| **42221** | response to chemical stimulus | 3.9729E–7 |
| **51247** | positive regulation of protein metabolic process | 4.5582E–7 |
| **31401** | positive regulation of protein modification process | 4.5582E–7 |
| **51246** | regulation of protein metabolic process | 4.7231E–7 |
| **8219** | cell death | 6.0996E–7 |
| **90066** | regulation of anatomical structure size | 6.3460E–7 |
| **16265** | death | 6.4982E–7 |
| **48519** | negative regulation of biological process | 7.7189E–7 |
| **43010** | camera-type eye development | 1.2266E–6 |
| **6950** | response to stress | 1.4899E–6 |
| **1836** | release of cytochrome c from mitochondria | 2.3185E–6 |
| **32535** | regulation of cellular component size | 4.9176E–6 |
| **42493** | response to drug | 9.1544E–6 |
| **1654** | eye development | 9.3503E–6 |
| **8361** | regulation of cell size | 9.6696E–6 |
| **65008** | regulation of biological quality | 9.9022E–6 |
| **43523** | regulation of neuron apoptosis | 1.1505E–5 |
| **8637** | apoptotic mitochondrial changes | 1.5474E–5 |
| **46668** | regulation of retinal cell programmed cell death | 1.5792E–5 |
| **51049** | regulation of transport | 1.7983E–5 |
| **9892** | negative regulation of metabolic process | 2.3064E–5 |
| **7005** | mitochondrion organization | 2.3841E–5 |
| **10033** | response to organic substance | 2.4464E–5 |
| **51239** | regulation of multicellular organismal process | 2.7010E–5 |
| **7423** | sensory organ development | 2.8008E–5 |
| **10604** | positive regulation of macromolecule metabolic process | 2.8008E–5 |

| | | |
|---|---|---|
| **9893** | positive regulation of metabolic process | 3.5530E–5 |
| **1558** | regulation of cell growth | 3.6071E–5 |
| **50896** | response to stimulus | 3.9011E–5 |
| **43065** | positive regulation of apoptosis | 3.9011E–5 |
| **9314** | response to radiation | 4.0362E–5 |
| **31325** | positive regulation of cellular metabolic process | 4.0362E–5 |
| **43068** | positive regulation of programmed cell death | 4.0362E–5 |
| **9607** | response to biotic stimulus | 4.0846E–5 |
| **60341** | regulation of cellular localization | 4.3170E–5 |
| **10942** | positive regulation of cell death | 4.3170E–5 |
| **40008** | regulation of growth | 5.6766E–5 |
| **6915** | apoptosis | 5.6766E–5 |
| **302** | response to reactive oxygen species | 5.6766E–5 |
| **10605** | negative regulation of macromolecule metabolic process | 5.7230E–5 |
| **51789** | response to protein stimulus | 5.9250E–5 |
| **51259** | protein oligomerization | 6.6096E–5 |
| **12501** | programmed cell death | 6.8345E–5 |
| **1934** | positive regulation of protein amino acid phosphorylation | 6.9774E–5 |
| **51881** | regulation of mitochondrial membrane potential | 8.4773E–5 |
| **43281** | regulation of caspase activity | 8.4773E–5 |
| **52548** | regulation of endopeptidase activity | 1.3401E–4 |
| **32879** | regulation of localization | 1.3401E–4 |
| **42327** | positive regulation of phosphorylation | 1.3401E–4 |
| **6508** | proteolysis | 1.4239E–4 |
| **45937** | positive regulation of phosphate metabolic process | 1.5387E–4 |
| **10562** | positive regulation of phosphorus metabolic process | 1.5387E–4 |
| **51094** | positive regulation of developmental process | 1.5387E–4 |
| **33554** | cellular response to stress | 1.6177E–4 |
| **52547** | regulation of peptidase activity | 1.7449E–4 |
| **1932** | regulation of protein amino acid phosphorylation | 1.9430E–4 |
| **7006** | mitochondrial membrane organization | 2.0786E–4 |
| **30307** | positive regulation of cell growth | 2.0909E–4 |
| **10039** | response to iron ion | 2.0909E–4 |
| **34097** | response to cytokine stimulus | 2.1716E–4 |
| **10646** | regulation of cell communication | 2.2072E–4 |
| **51130** | positive regulation of cellular component organization | 2.2347E–4 |
| **6917** | induction of apoptosis | 2.3339E–4 |
| **22402** | cell cycle process | 2.3339E–4 |
| **9416** | response to light stimulus | 2.3547E–4 |
| **12502** | induction of programmed cell death | 2.3576E–4 |
| **6979** | response to oxidative stress | 2.4764E–4 |
| **43524** | negative regulation of neuron apoptosis | 2.6452E–4 |
| **51128** | regulation of cellular component organization | 3.0405E–4 |
| **45793** | positive regulation of cell size | 3.2010E–4 |
| **51704** | multi-organism process | 3.2010E–4 |
| **43933** | macromolecular complex subunit organization | 3.3609E–4 |
| **278** | mitotic cell cycle | 3.3609E–4 |
| **50789** | regulation of biological process | 3.3609E–4 |

| | | |
|---|---|---|
| **51716** | cellular response to stimulus | 3.4538E–4 |
| **45595** | regulation of cell differentiation | 4.0199E–4 |
| **77** | DNA damage checkpoint | 4.4934E–4 |
| **3008** | system process | 4.5090E–4 |
| **51726** | regulation of cell cycle | 4.5180E–4 |
| **51412** | response to corticosterone stimulus | 4.8546E–4 |
| **51051** | negative regulation of transport | 4.8546E–4 |
| **70271** | protein complex biogenesis | 4.9255E–4 |
| **6461** | protein complex assembly | 4.9255E–4 |
| **60284** | regulation of cell development | 5.3505E–4 |
| **9411** | response to UV | 5.5304E–4 |
| **43086** | negative regulation of catalytic activity | 5.6912E–4 |
| **31570** | DNA integrity checkpoint | 5.9515E–4 |
| **45597** | positive regulation of cell differentiation | 6.1827E–4 |
| **51248** | negative regulation of protein metabolic process | 6.7369E–4 |
| **9636** | response to toxin | 6.8659E–4 |
| **42542** | response to hydrogen peroxide | 6.8659E–4 |
| **10647** | positive regulation of cell communication | 7.0631E–4 |
| **50790** | regulation of catalytic activity | 7.2451E–4 |
| **22607** | cellular component assembly | 8.0667E–4 |
| **65007** | biological regulation | 8.9663E–4 |
| **8635** | activation of caspase activity by cytochrome c | 8.9663E–4 |
| **31400** | negative regulation of protein modification process | 8.9663E–4 |
| **50793** | regulation of developmental process | 9.3990E–4 |
| **51385** | response to mineralocorticoid stimulus | 9.5287E–4 |
| **44092** | negative regulation of molecular function | 9.8814E–4 |
| **48545** | response to steroid hormone stimulus | 9.9975E–4 |
| **30308** | negative regulation of cell growth | 9.9975E–4 |
| **7626** | locomotory behavior | 1.0103E–3 |
| **35466** | regulation of signaling pathway | 1.0379E–3 |
| **51260** | protein homooligomerization | 1.0379E–3 |
| **50794** | regulation of cellular process | 1.0414E–3 |
| **51240** | positive regulation of multicellular organismal process | 1.0773E–3 |
| **9605** | response to external stimulus | 1.0773E–3 |
| **35468** | positive regulation of signaling pathway | 1.0809E–3 |
| **9719** | response to endogenous stimulus | 1.0869E–3 |
| **65003** | macromolecular complex assembly | 1.0889E–3 |
| **46902** | regulation of mitochondrial membrane permeability | 1.1765E–3 |
| **226** | microtubule cytoskeleton organization | 1.1792E–3 |
| **44265** | cellular macromolecule catabolic process | 1.1920E–3 |
| **6996** | organelle organization | 1.1920E–3 |
| **8624** | induction of apoptosis by extracellular signals | 1.2131E–3 |
| **45471** | response to ethanol | 1.3433E–3 |
| **45792** | negative regulation of cell size | 1.3433E–3 |
| **7010** | cytoskeleton organization | 1.3676E–3 |
| **30163** | protein catabolic process | 1.4035E–3 |
| **10243** | response to organic nitrogen | 1.4126E–3 |
| **44085** | cellular component biogenesis | 1.4282E–3 |

| | | |
|---|---|---|
| **30264** | nuclear fragmentation involved in apoptotic nuclear change | 1.4282E–3 |
| **46670** | positive regulation of retinal cell programmed cell death | 1.4282E–3 |
| **71763** | nuclear membrane organization | 1.4282E–3 |
| **60732** | positive regulation of inositol phosphate biosynthetic process | 1.4282E–3 |
| **43627** | response to estrogen stimulus | 1.5801E–3 |
| **42325** | regulation of phosphorylation | 1.6492E–3 |
| **8284** | positive regulation of cell proliferation | 1.6492E–3 |
| **32269** | negative regulation of cellular protein metabolic process | 1.7003E–3 |
| **48678** | response to axon injury | 1.7342E–3 |
| **7017** | microtubule-based process | 1.7374E–3 |
| **35239** | tube morphogenesis | 1.7986E–3 |
| **7610** | behavior | 1.8708E–3 |
| **22603** | regulation of anatomical structure morphogenesis | 1.9134E–3 |
| **65009** | regulation of molecular function | 1.9385E–3 |
| **51707** | response to other organism | 1.9846E–3 |
| **10720** | positive regulation of cell development | 2.0471E–3 |
| **45927** | positive regulation of growth | 2.1720E–3 |
| **7049** | cell cycle | 2.2484E–3 |
| **9611** | response to wounding | 2.4096E–3 |
| **51603** | proteolysis involved in cellular protein catabolic process | 2.4327E–3 |
| **31103** | axon regeneration | 2.4327E–3 |
| **32387** | negative regulation of intracellular transport | 2.4327E–3 |
| **51174** | regulation of phosphorus metabolic process | 2.4327E–3 |
| **19220** | regulation of phosphate metabolic process | 2.4327E–3 |
| **44057** | regulation of system process | 2.4993E–3 |
| **44257** | cellular protein catabolic process | 2.5543E–3 |
| **45926** | negative regulation of growth | 2.5677E–3 |
| **9991** | response to extracellular stimulus | 2.6650E–3 |
| **6997** | nucleus organization | 2.6650E–3 |
| **43085** | positive regulation of catalytic activity | 2.9188E–3 |
| **32880** | regulation of protein localization | 2.9642E–3 |
| **19217** | regulation of fatty acid metabolic process | 3.0738E–3 |
| **16043** | cellular component organization | 3.1605E–3 |
| **42770** | DNA damage response, signal transduction | 3.2152E–3 |
| **9725** | response to hormone stimulus | 3.4533E–3 |
| **90199** | regulation of release of cytochrome c from mitochondria | 3.4688E–3 |
| **10919** | regulation of inositol phosphate biosynthetic process | 3.4688E–3 |
| **9057** | macromolecule catabolic process | 3.5246E–3 |
| **31102** | neuron projection regeneration | 3.5851E–3 |
| **51436** | negative regulation of ubiquitin-protein ligase activity involved in mitotic cell cycle | 3.7131E–3 |
| **32386** | regulation of intracellular transport | 3.8874E–3 |
| **31145** | anaphase-promoting complex-dependent proteasomal ubiquitin-dependent protein catabolic process | 3.9594E–3 |
| **23056** | positive regulation of signaling process | 4.0498E–3 |
| **51437** | positive regulation of ubiquitin-protein ligase activity involved in mitotic cell cycle | 4.2169E–3 |
| **44248** | cellular catabolic process | 4.2492E–3 |

| | | |
|---|---|---|
| **32502** | developmental process | 4.2492E–3 |
| **9653** | anatomical structure morphogenesis | 4.7128E–3 |
| **51352** | negative regulation of ligase activity | 4.7165E–3 |
| **51444** | negative regulation of ubiquitin-protein ligase activity | 4.7165E–3 |
| **6986** | response to unfolded protein | 4.7165E–3 |
| **819** | sister chromatid segregation | 4.7421E–3 |
| **31324** | negative regulation of cellular metabolic process | 4.7762E–3 |
| **51329** | interphase of mitotic cell cycle | 4.7837E–3 |
| **46686** | response to cadmium ion | 4.9962E–3 |
| **51402** | neuron apoptosis | 4.9962E–3 |
| **9056** | catabolic process | 5.1147E–3 |
| **51223** | regulation of protein transport | 5.1147E–3 |
| **43170** | macromolecule metabolic process | 5.1147E–3 |
| **51046** | regulation of secretion | 5.1147E–3 |
| **51439** | regulation of ubiquitin-protein ligase activity involved in mitotic cell cycle | 5.1501E–3 |
| **48878** | chemical homeostasis | 5.1909E–3 |
| **33043** | regulation of organelle organization | 5.3521E–3 |
| **31667** | response to nutrient levels | 5.4875E–3 |
| **51325** | interphase | 5.5629E–3 |
| **35295** | tube development | 5.5967E–3 |
| **46688** | response to copper ion | 5.6787E–3 |
| **51443** | positive regulation of ubiquitin-protein ligase activity | 5.6787E–3 |
| **48856** | anatomical structure development | 5.6787E–3 |
| **75** | cell cycle checkpoint | 5.6836E–3 |
| **14070** | response to organic cyclic substance | 5.6836E–3 |
| **70141** | response to UV-A | 5.7084E–3 |
| **14012** | axon regeneration in the peripheral nervous system | 5.7084E–3 |
| **43497** | regulation of protein heterodimerization activity | 5.7084E–3 |
| **60561** | apoptosis involved in morphogenesis | 5.7084E–3 |
| **7178** | transmembrane receptor protein serine/threonine kinase signaling pathway | 5.8328E–3 |
| **43623** | cellular protein complex assembly | 5.9856E–3 |
| **7154** | cell communication | 6.1286E–3 |
| **70201** | regulation of establishment of protein localization | 6.3399E–3 |
| **737** | DNA catabolic process, endonucleolytic | 6.3399E–3 |
| **70997** | neuron death | 6.3399E–3 |
| **6309** | DNA fragmentation involved in apoptotic nuclear change | 6.3399E–3 |
| **51351** | positive regulation of ligase activity | 6.4437E–3 |
| **10629** | negative regulation of gene expression | 7.0016E–3 |
| **9267** | cellular response to starvation | 7.2401E–3 |
| **6873** | cellular ion homeostasis | 7.5724E–3 |
| **30534** | adult behavior | 7.6136E–3 |
| **51641** | cellular localization | 7.8168E–3 |
| **9987** | cellular process | 8.0794E–3 |
| **22403** | cell cycle phase | 8.2496E–3 |
| **30509** | BMP signaling pathway | 8.3094E–3 |
| **46928** | regulation of neurotransmitter secretion | 8.3094E–3 |
| **43525** | positive regulation of neuron apoptosis | 8.3094E–3 |
| **31397** | negative regulation of protein ubiquitination | 8.3094E–3 |

| | | |
|---|---|---|
| **55082** | cellular chemical homeostasis | 8.3094E–3 |
| **32847** | regulation of cellular pH reduction | 8.4005E–3 |
| **71681** | cellular response to indole-3-methanol | 8.4005E–3 |
| **71680** | response to indole-3-methanol | 8.4005E–3 |
| **2326** | B cell lineage commitment | 8.4005E–3 |
| **2360** | T cell lineage commitment | 8.4005E–3 |
| **10821** | regulation of mitochondrion organization | 8.4005E–3 |
| **7021** | tubulin complex assembly | 8.4005E–3 |
| **48608** | reproductive structure development | 8.4101E–3 |
| **1541** | ovarian follicle development | 8.6387E–3 |
| **42592** | homeostatic process | 8.7978E–3 |
| **44093** | positive regulation of molecular function | 8.9894E–3 |
| **42127** | regulation of cell proliferation | 8.9894E–3 |
| **45913** | positive regulation of carbohydrate metabolic process | 8.9894E–3 |
| **43154** | negative regulation of caspase activity | 8.9894E–3 |
| **10676** | positive regulation of cellular carbohydrate metabolic process | 8.9894E–3 |
| **31396** | regulation of protein ubiquitination | 9.1271E–3 |
| **51438** | regulation of ubiquitin-protein ligase activity | 9.1271E–3 |
| **10565** | regulation of cellular ketone metabolic process | 9.1271E–3 |
| **50953** | sensory perception of light stimulus | 9.3559E–3 |
| **7601** | visual perception | 9.3559E–3 |
| **3012** | muscle system process | 9.5278E–3 |
| **23034** | intracellular signaling pathway | 9.9988E–3 |
| **51224** | negative regulation of protein transport | 1.0380E–2 |
| **48729** | tissue morphogenesis | 1.0422E–2 |
| **51340** | regulation of ligase activity | 1.0465E–2 |
| **10740** | positive regulation of intracellular protein kinase cascade | 1.0567E–2 |
| **9967** | positive regulation of signal transduction | 1.0567E–2 |
| **6511** | ubiquitin-dependent protein catabolic process | 1.0795E–2 |
| **8344** | adult locomotory behavior | 1.0947E–2 |
| **43122** | regulation of I-kappaB kinase/NF-kappaB cascade | 1.1056E–2 |
| **46889** | positive regulation of lipid biosynthetic process | 1.1056E–2 |
| **48538** | thymus development | 1.1056E–2 |
| **8634** | negative regulation of survival gene product expression | 1.1225E–2 |
| **48739** | cardiac muscle fiber development | 1.1225E–2 |
| **32469** | endoplasmic reticulum calcium ion homeostasis | 1.1225E–2 |
| **8406** | gonad development | 1.1317E–2 |
| **70887** | cellular response to chemical stimulus | 1.1601E–2 |
| **45786** | negative regulation of cell cycle | 1.1690E–2 |
| **43632** | modification-dependent macromolecule catabolic process | 1.1690E–2 |
| **19941** | modification-dependent protein catabolic process | 1.1690E–2 |
| **19538** | protein metabolic process | 1.1995E–2 |
| **30262** | apoptotic nuclear change | 1.2096E–2 |
| **19725** | cellular homeostasis | 1.2322E–2 |
| **50801** | ion homeostasis | 1.2989E–2 |
| **48754** | branching morphogenesis of a tube | 1.3229E–2 |
| **19216** | regulation of lipid metabolic process | 1.3268E–2 |
| **33077** | T cell differentiation in the thymus | 1.3268E–2 |

| | | |
|---|---|---|
| **43255** | regulation of carbohydrate biosynthetic process | 1.3268E–2 |
| **10595** | positive regulation of endothelial cell migration | 1.3268E–2 |
| **51588** | regulation of neurotransmitter transport | 1.3268E–2 |
| **34599** | cellular response to oxidative stress | 1.3482E–2 |
| **7612** | learning | 1.4385E–2 |
| **45165** | cell fate commitment | 1.4521E–2 |
| **85** | G2 phase of mitotic cell cycle | 1.4541E–2 |
| **51319** | G2 phase | 1.4541E–2 |
| **10332** | response to gamma radiation | 1.4590E–2 |
| **46890** | regulation of lipid biosynthetic process | 1.5117E–2 |
| **34621** | cellular macromolecular complex subunit organization | 1.5532E–2 |
| **48285** | organelle fission | 1.5728E–2 |
| **43161** | proteasomal ubiquitin-dependent protein catabolic process | 1.5728E–2 |
| **10498** | proteasomal protein catabolic process | 1.5728E–2 |
| **51969** | regulation of transmission of nerve impulse | 1.5728E–2 |
| **31398** | positive regulation of protein ubiquitination | 1.5763E–2 |
| **42594** | response to starvation | 1.5763E–2 |
| **6921** | cellular component disassembly involved in apoptosis | 1.5813E–2 |
| **31668** | cellular response to extracellular stimulus | 1.6369E–2 |
| **51346** | negative regulation of hydrolase activity | 1.6642E–2 |
| **71496** | cellular response to external stimulus | 1.6962E–2 |
| **51384** | response to glucocorticoid stimulus | 1.6962E–2 |
| **48869** | cellular developmental process | 1.6993E–2 |
| **9409** | response to cold | 1.7077E–2 |
| **1658** | branching involved in ureteric bud morphogenesis | 1.7077E–2 |
| **10466** | negative regulation of peptidase activity | 1.7077E–2 |
| **60688** | regulation of morphogenesis of a branching structure | 1.7077E–2 |
| **50808** | synapse organization | 1.7156E–2 |
| **6919** | activation of caspase activity | 1.7156E–2 |
| **40014** | regulation of multicellular organism growth | 1.7156E–2 |
| **10623** | developmental programmed cell death | 1.7646E–2 |
| **31109** | microtubule polymerization or depolymerization | 1.7646E–2 |
| **43496** | regulation of protein homodimerization activity | 1.7646E–2 |
| **60052** | neurofilament cytoskeleton organization | 1.7646E–2 |
| **44238** | primary metabolic process | 1.7646E–2 |
| **48468** | cell development | 1.8062E–2 |
| **7568** | aging | 1.8113E–2 |
| **48731** | system development | 1.8226E–2 |
| **61138** | morphogenesis of a branching epithelium | 1.8352E–2 |
| **30003** | cellular cation homeostasis | 1.8505E–2 |
| **45137** | development of primary sexual characteristics | 1.8505E–2 |
| **23051** | regulation of signaling process | 1.8505E–2 |
| **7275** | multicellular organismal development | 1.8558E–2 |
| **8584** | male gonad development | 1.8558E–2 |
| **60675** | ureteric bud morphogenesis | 1.9728E–2 |
| **1666** | response to hypoxia | 2.0108E–2 |
| **31644** | regulation of neurological system process | 2.0108E–2 |
| **48646** | anatomical structure formation involved in morphogenesis | 2.0108E–2 |

| | | |
|---|---|---|
| **1894** | tissue homeostasis | 2.0467E–2 |
| **32355** | response to estradiol stimulus | 2.0467E–2 |
| **6954** | inflammatory response | 2.0467E–2 |
| **34622** | cellular macromolecular complex assembly | 2.0782E–2 |
| **51649** | establishment of localization in cell | 2.0782E–2 |
| **31960** | response to corticosteroid stimulus | 2.0790E–2 |
| **60** | protein import into nucleus, translocation | 2.0879E–2 |
| **50772** | positive regulation of axonogenesis | 2.0879E–2 |
| **1782** | B cell homeostasis | 2.0879E–2 |
| **1963** | synaptic transmission, dopaminergic | 2.0879E–2 |
| **6878** | cellular copper ion homeostasis | 2.0879E–2 |
| **6998** | nuclear envelope organization | 2.0879E–2 |
| **30154** | cell differentiation | 2.0888E–2 |
| **48534** | hemopoietic or lymphoid organ development | 2.1056E–2 |
| **60255** | regulation of macromolecule metabolic process | 2.1667E–2 |
| **16044** | cellular membrane organization | 2.1888E–2 |
| **61024** | membrane organization | 2.2235E–2 |
| **2009** | morphogenesis of an epithelium | 2.2270E–2 |
| **1776** | leukocyte homeostasis | 2.2270E–2 |
| **6936** | muscle contraction | 2.2270E–2 |
| **10627** | regulation of intracellular protein kinase cascade | 2.2834E–2 |
| **10212** | response to ionizing radiation | 2.2834E–2 |
| **51651** | maintenance of location in cell | 2.2834E–2 |
| **40007** | growth | 2.3153E–2 |
| **9891** | positive regulation of biosynthetic process | 2.3530E–2 |
| **8306** | associative learning | 2.3840E–2 |
| **34637** | cellular carbohydrate biosynthetic process | 2.3840E–2 |
| **70482** | response to oxygen levels | 2.4506E–2 |
| **32501** | multicellular organismal process | 2.4506E–2 |
| **70306** | lens fiber cell differentiation | 2.4506E–2 |
| **55070** | copper ion homeostasis | 2.4506E–2 |
| **32026** | response to magnesium ion | 2.4506E–2 |
| **43280** | positive regulation of caspase activity | 2.4665E–2 |
| **10952** | positive regulation of peptidase activity | 2.4665E–2 |
| **7416** | synapse assembly | 2.5349E–2 |
| **48513** | organ development | 2.7020E–2 |
| **46474** | glycerophospholipid biosynthetic process | 2.7020E–2 |
| **50769** | positive regulation of neurogenesis | 2.7020E–2 |
| **7611** | learning or memory | 2.7020E–2 |
| **9790** | embryonic development | 2.7020E–2 |
| **7569** | cell aging | 2.7020E–2 |
| **48747** | muscle fiber development | 2.7020E–2 |
| **2520** | immune system development | 2.7888E–2 |
| **60429** | epithelium development | 2.8392E–2 |
| **43123** | positive regulation of I-kappaB kinase/NF-kappaB cascade | 2.8403E–2 |
| **45723** | positive regulation of fatty acid biosynthetic process | 2.8403E–2 |
| **6107** | oxaloacetate metabolic process | 2.8403E–2 |
| **7080** | mitotic metaphase plate congression | 2.8403E–2 |

| | | |
|---|---|---|
| **48070** | regulation of developmental pigmentation | 2.8403E–2 |
| **19222** | regulation of metabolic process | 2.8403E–2 |
| **70** | mitotic sister chromatid segregation | 2.8585E–2 |
| **8585** | female gonad development | 2.8850E–2 |
| **22602** | ovulation cycle process | 3.0225E–2 |
| **46651** | lymphocyte proliferation | 3.0654E–2 |
| **8152** | metabolic process | 3.1887E–2 |
| **42110** | T cell activation | 3.2161E–2 |
| **7167** | enzyme linked receptor protein signaling pathway | 3.2517E–2 |
| **34614** | cellular response to reactive oxygen species | 3.2635E–2 |
| **6839** | mitochondrial transport | 3.2635E–2 |
| **45736** | negative regulation of cyclin-dependent protein kinase activity | 3.2635E–2 |
| **42100** | B cell proliferation | 3.2635E–2 |
| **7617** | mating behavior | 3.2635E–2 |
| **32225** | regulation of synaptic transmission, dopaminergic | 3.2635E–2 |
| **55080** | cation homeostasis | 3.2635E–2 |
| **51960** | regulation of nervous system development | 3.3361E–2 |
| **1763** | morphogenesis of a branching structure | 3.3472E–2 |
| **60627** | regulation of vesicle-mediated transport | 3.3472E–2 |
| **46546** | development of primary male sexual characteristics | 3.3472E–2 |
| **31669** | cellular response to nutrient levels | 3.3472E–2 |
| **32943** | mononuclear cell proliferation | 3.4146E–2 |
| **9966** | regulation of signal transduction | 3.4756E–2 |
| **23033** | signaling pathway | 3.4767E–2 |
| **32989** | cellular component morphogenesis | 3.4821E–2 |
| **51241** | negative regulation of multicellular organismal process | 3.4821E–2 |
| **7346** | regulation of mitotic cell cycle | 3.4821E–2 |
| **9617** | response to bacterium | 3.5610E–2 |
| **7548** | sex differentiation | 3.5610E–2 |
| **46545** | development of primary female sexual characteristics | 3.5683E–2 |
| **90305** | nucleic acid phosphodiester bond hydrolysis | 3.5683E–2 |
| **45833** | negative regulation of lipid metabolic process | 3.5683E–2 |
| **70661** | leukocyte proliferation | 3.5683E–2 |
| **280** | nuclear division | 3.5719E–2 |
| **7067** | mitosis | 3.5719E–2 |
| **14059** | regulation of dopamine secretion | 3.5719E–2 |
| **51310** | metaphase plate congression | 3.5719E–2 |
| **10559** | regulation of glycoprotein biosynthetic process | 3.5719E–2 |
| **43536** | positive regulation of blood vessel endothelial cell migration | 3.5719E–2 |
| **6833** | water transport | 3.5719E–2 |
| **31571** | G1/S DNA damage checkpoint | 3.5719E–2 |
| **60749** | mammary gland alveolus development | 3.5719E–2 |
| **8088** | axon cargo transport | 3.5719E–2 |
| **32729** | positive regulation of interferon-gamma production | 3.5719E–2 |
| **45321** | leukocyte activation | 3.5719E–2 |
| **30097** | hemopoiesis | 3.5719E–2 |
| **33157** | regulation of intracellular protein transport | 3.5934E–2 |
| **31099** | regeneration | 3.5934E–2 |

| | | |
|---|---|---|
| **209** | protein polyubiquitination | 3.6744E–2 |
| **3006** | reproductive developmental process | 3.6744E–2 |
| **60562** | epithelial tube morphogenesis | 3.6744E–2 |
| **42698** | ovulation cycle | 3.7120E–2 |
| **7369** | gastrulation | 3.7120E–2 |
| **22008** | neurogenesis | 3.7418E–2 |
| **2237** | response to molecule of bacterial origin | 3.7531E–2 |
| **2521** | leukocyte differentiation | 3.7531E–2 |
| **10594** | regulation of endothelial cell migration | 3.8592E–2 |
| **30335** | positive regulation of cell migration | 3.8592E–2 |
| **3013** | circulatory system process | 3.8829E–2 |
| **8015** | blood circulation | 3.8829E–2 |
| **87** | M phase of mitotic cell cycle | 3.9248E–2 |
| **71310** | cellular response to organic substance | 3.9248E–2 |
| **50804** | regulation of synaptic transmission | 3.9248E–2 |
| **33138** | positive regulation of peptidyl-serine phosphorylation | 3.9248E–2 |
| **8625** | induction of apoptosis via death domain receptors | 3.9248E–2 |
| **45773** | positive regulation of axon extension | 3.9248E–2 |
| **42044** | fluid transport | 3.9248E–2 |
| **32330** | regulation of chondrocyte differentiation | 3.9248E–2 |
| **10675** | regulation of cellular carbohydrate metabolic process | 4.0076E–2 |
| **6066** | alcohol metabolic process | 4.0076E–2 |
| **31328** | positive regulation of cellular biosynthetic process | 4.0076E–2 |
| **904** | cell morphogenesis involved in differentiation | 4.0076E–2 |
| **46660** | female sex differentiation | 4.0076E–2 |
| **46661** | male sex differentiation | 4.0076E–2 |
| **7059** | chromosome segregation | 4.0076E–2 |
| **45017** | glycerolipid biosynthetic process | 4.0076E–2 |
| **42391** | regulation of membrane potential | 4.1530E–2 |
| **30183** | B cell differentiation | 4.1530E–2 |
| **50679** | positive regulation of epithelial cell proliferation | 4.1530E–2 |
| **6109** | regulation of carbohydrate metabolic process | 4.1530E–2 |
| **46649** | lymphocyte activation | 4.1530E–2 |
| **51222** | positive regulation of protein transport | 4.1530E–2 |
| **48871** | multicellular organismal homeostasis | 4.1530E–2 |
| **43434** | response to peptide hormone stimulus | 4.1530E–2 |
| **9651** | response to salt stress | 4.1530E–2 |
| **14048** | regulation of glutamate secretion | 4.1530E–2 |
| **10165** | response to X-ray | 4.1530E–2 |
| **51354** | negative regulation of oxidoreductase activity | 4.1530E–2 |
| **22414** | reproductive process | 4.1530E–2 |
| **48609** | reproductive process in a multicellular organism | 4.1530E–2 |
| **32504** | multicellular organism reproduction | 4.1530E–2 |
| **51235** | maintenance of location | 4.1530E–2 |
| **50877** | neurological system process | 4.1530E–2 |
| **16567** | protein ubiquitination | 4.1530E–2 |
| **51336** | regulation of hydrolase activity | 4.1530E–2 |
| **3** | reproduction | 4.1530E–2 |

| | | |
|---|---|---|
| **7267** | cell-cell signaling | 4.1530E–2 |
| **6259** | DNA metabolic process | 4.1530E–2 |
| **51272** | positive regulation of cellular component movement | 4.1530E–2 |
| **40017** | positive regulation of locomotion | 4.1530E–2 |
| **32848** | negative regulation of cellular pH reduction | 4.1530E–2 |
| **90200** | positive regulation of release of cytochrome c from mitochondria | 4.1530E–2 |
| **32976** | release of matrix enzymes from mitochondria | 4.1530E–2 |
| **70495** | negative regulation of thrombin receptor signaling pathway | 4.1530E–2 |
| **70494** | regulation of thrombin receptor signaling pathway | 4.1530E–2 |
| **33693** | neurofilament bundle assembly | 4.1530E–2 |
| **21747** | cochlear nucleus development | 4.1530E–2 |
| **46370** | fructose biosynthetic process | 4.1530E–2 |
| **50747** | positive regulation of lipoprotein metabolic process | 4.1530E–2 |
| **46674** | induction of retinal programmed cell death | 4.1530E–2 |
| **50812** | regulation of acyl-CoA biosynthetic process | 4.1530E–2 |
| **1711** | endodermal cell fate commitment | 4.1530E–2 |
| **14041** | regulation of neuron maturation | 4.1530E–2 |
| **14042** | positive regulation of neuron maturation | 4.1530E–2 |
| **6060** | sorbitol metabolic process | 4.1530E–2 |
| **6062** | sorbitol catabolic process | 4.1530E–2 |
| **51124** | synaptic growth at neuromuscular junction | 4.1530E–2 |
| **51160** | L-xylitol catabolic process | 4.1530E–2 |
| **51164** | L-xylitol metabolic process | 4.1530E–2 |
| **42999** | regulation of Golgi to plasma membrane CFTR protein transport | 4.1530E–2 |
| **43002** | negative regulation of Golgi to plasma membrane CFTR protein transport | 4.1530E–2 |
| **43004** | cytoplasmic sequestering of CFTR protein | 4.1530E–2 |
| **51315** | attachment of spindle microtubules to kinetochore involved in mitotic sister chromatid segregation | 4.1530E–2 |
| **43217** | myelin maintenance | 4.1530E–2 |
| **43369** | CD4-positive or CD8-positive, alpha-beta T cell lineage commitment | 4.1530E–2 |
| **43375** | CD8-positive, alpha-beta T cell lineage commitment | 4.1530E–2 |
| **51581** | negative regulation of neurotransmitter uptake | 4.1530E–2 |
| **51585** | negative regulation of dopamine uptake | 4.1530E–2 |
| **51611** | regulation of serotonin uptake | 4.1530E–2 |
| **51612** | negative regulation of serotonin uptake | 4.1530E–2 |
| **51621** | regulation of norepinephrine uptake | 4.1530E–2 |
| **51622** | negative regulation of norepinephrine uptake | 4.1530E–2 |
| **2477** | antigen processing and presentation of exogenous peptide antigen via MHC class Ib | 4.1530E–2 |
| **2481** | antigen processing and presentation of exogenous protein antigen via MHC class Ib, TAP-dependent | 4.1530E–2 |
| **2525** | acute inflammatory response to non-antigenic stimulus | 4.1530E–2 |
| **2575** | basophil chemotaxis | 4.1530E–2 |
| **14916** | regulation of lung blood pressure | 4.1530E–2 |
| **10822** | positive regulation of mitochondrion organization | 4.1530E–2 |
| **6808** | regulation of nitrogen utilization | 4.1530E–2 |
| **51913** | regulation of synaptic plasticity by chemical substance | 4.1530E–2 |
| **51914** | positive regulation of synaptic plasticity by chemical substance | 4.1530E–2 |

| | | |
|---|---|---|
| **51915** | induction of synaptic plasticity by chemical substance | 4.1530E–2 |
| **51945** | negative regulation of catecholamine uptake involved in synaptic transmission | 4.1530E–2 |
| **60215** | primitive hemopoiesis | 4.1530E–2 |
| **60266** | negative regulation of respiratory burst involved in inflammatory response | 4.1530E–2 |
| **60268** | negative regulation of respiratory burst | 4.1530E–2 |
| **60319** | primitive erythrocyte differentiation | 4.1530E–2 |
| **19407** | hexitol catabolic process | 4.1530E–2 |
| **19519** | pentitol metabolic process | 4.1530E–2 |
| **19527** | pentitol catabolic process | 4.1530E–2 |
| **31987** | locomotion involved in locomotory behavior | 4.1530E–2 |
| **60697** | positive regulation of phospholipid catabolic process | 4.1530E–2 |
| **48668** | collateral sprouting | 4.1530E–2 |
| **48669** | collateral sprouting in the absence of injury | 4.1530E–2 |
| **32287** | myelin maintenance in the peripheral nervous system | 4.1530E–2 |
| **48743** | positive regulation of skeletal muscle fiber development | 4.1530E–2 |
| **32621** | interleukin-18 production | 4.1530E–2 |
| **16238** | chaperone-mediated autophagy | 4.1530E–2 |
| **46907** | intracellular transport | 4.1908E–2 |
| **44262** | cellular carbohydrate metabolic process | 4.2121E–2 |
| **16486** | peptide hormone processing | 4.2148E–2 |
| **19098** | reproductive behavior | 4.2148E–2 |
| **60395** | SMAD protein signal transduction | 4.2148E–2 |
| **48732** | gland development | 4.2359E–2 |
| **55002** | striated muscle cell development | 4.4786E–2 |
| **9615** | response to virus | 4.5859E–2 |
| **10638** | positive regulation of organelle organization | 4.5859E–2 |
| **50000** | chromosome localization | 4.6700E–2 |
| **30104** | water homeostasis | 4.6700E–2 |
| **51303** | establishment of chromosome localization | 4.6700E–2 |
| **10862** | positive regulation of pathway-restricted SMAD protein phosphorylation | 4.6700E–2 |
| **61035** | regulation of cartilage development | 4.6700E–2 |
| **45944** | positive regulation of transcription from RNA polymerase II promoter | 4.6710E–2 |
| **10769** | regulation of cell morphogenesis involved in differentiation | 4.6914E–2 |
| **90092** | regulation of transmembrane receptor protein serine/threonine kinase signaling pathway | 4.6914E–2 |
| **48593** | camera-type eye morphogenesis | 4.8988E–2 |
| **48638** | regulation of developmental growth | 4.8988E–2 |
| **1817** | regulation of cytokine production | 4.9769E–2 |
| **48666** | neuron development | 4.9918E–2 |

All biological processes. Significance level $p < 0.05$.
